# Fitness Training for γδ T cells in mouse and human atherosclerosis takes place in plaques and artery tertiary lymphoid organs

**DOI:** 10.1186/s13073-026-01613-1

**Published:** 2026-02-24

**Authors:** Zhihua Wang, Ting Sun, Yixin Zhang, Yutao Li, Chuankai Zhang, Xinwen Dou, Xinyi Deng, Zhipeng Li, Jingpu Zhu, Mingyang Hong, Yi Ran, Shu Wang, Liping Li, Junjie Zheng, Cong Wen, Xi Zhang, Shu Lu, Sagar, Sarajo K. Mohanta, Andreas J.R. Habenicht, Changjun Yin

**Affiliations:** 1 Division of Vascular Surgery, The First Affiliated Hospital of Sun Yat-sen University, Guangzhou, China; 2 Institute for Cardiovascular Prevention (IPEK), Ludwig-Maximilians-University (LMU), Munich, Germany; 3 Institution of Precision Medicine, The First Affiliated Hospital of Sun Yat-sen University, Guangzhou, China; 4 Nanjing Key Laboratory for Cardiovascular Information and Health Engineering Medicine, Institute of Clinical Medicine, Cardiovascular Medical Center, Medical School, Nanjing Drum Tower Hospital, Nanjing University, Nanjing, 210093 China; 5 Department of Oncology, Cancer Center, The First Affiliated Hospital of Sun Yat-sen University, Guangzhou, China; 6 DZHK (German Centre for Cardiovascular Research), Partner site Munich Heart Alliance, Munich, Germany; 7 Institute of Intelligent Sport and Proactive Health, Department of Health and Physical Education, Jianghan University, Wuhan, China; 8 Department of Vascular Surgery, Renji Hospital, School of Medicine, Shanghai Jiao Tong University, Shanghai, China; 9 Department of Medicine II (Gastroenterology, Hepatology, Endocrinology, and Infectious Diseases), Faculty of Medicine, Freiburg University Medical Center, University of Freiburg, Freiburg, Germany; 10 Easemedcontrol R&D, Schraudolphstraße 5, 80799 München, Germany; 11 NHC Key Laboratory of Assisted Circulation and Vascular Diseases, Sun Yat-sen University, Guangzhou, China

**Keywords:** γδ T cells, atherosclerosis, immune education, γδ T17 cells, single-cell RNA sequencing.

## Abstract

**Background:**

γδ T cells represent a heterogeneous family of innate-like lymphocytes with adaptive immunity features. Although abundant in barrier tissues such as skin and intestine, γδ T cells are rare in the cardiovascular system, which severely limits exploration of their roles in atherosclerosis. Consequently, the spatial localization, functional states, and antigen-driven responses of γδ T cells within atherosclerotic lesions remain poorly defined.

**Methods:**

To nevertheless examine γδ T cells in atherosclerosis, we employed an integrative, three-pronged strategy combining γδ T cell single-cell RNA sequencing (scRNA-seq) and single-cell T cell receptor sequencing (scTCR-seq), spatial transcriptomics, and large-scale multi-dataset scRNA-seq integration across multiple hyperlipidemic mouse models. Large-scale multi-dataset scRNA-seq integration was also applied to human atherosclerotic plaque datasets.

**Results:**

We found significant enrichment of γδ T cells within both atherosclerotic plaques and artery tertiary lymphoid organs (ATLOs), particularly of the proinflammatory IL17-producing γδ T17 subtype. Unexpectedly, γδ T17 cells containing paired Vγ6Vδ4 TCR chains with identical complementarity-determining region 3 (CDR3) sequences underwent clonal expansion in atherosclerotic plaques and ATLOs. Transcriptomic analysis revealed that plaques and ATLOs locally educate γδ T17 cells - here termed γδ eduT17 cells - towards an anti-apoptotic, tissue-resident, and apparent hypofunctional phenotype. Furthermore, γδ eduT17 cells underwent metabolic reprogramming within the atherosclerotic microenvironment. Spatial transcriptomics revealed that γδ T cells preferentially localize in ATLOs, particularly at the interface between T cell zones and B cell follicles. Multi-dataset integration confirmed the conservation of these features across multiple hyperlipidemic mouse models. In contrast, human atherosclerotic plaques harbored substantially fewer γδ T cells and the human phenotypes were dominated by an effector/cytolytic γδ T cell subtype, characterized by transcriptomes enriched in cytotoxic effector molecules.

**Conclusions:**

Our findings identify γδ T cells as a previously underappreciated T cell lineage population in atherosclerosis. Murine atherosclerosis is characterized by the enrichment, education, and clonal expansion of proinflammatory γδ eduT17 cells within plaques and ATLOs. In contrast, human plaques harbor γδ T cells with cytolytic features, suggesting divergent roles of γδ T cells between species. These results highlight the importance of local vascular microenvironments in shaping γδ T cell function and emphasize the need for caution when extrapolating mechanistic insights from mouse models to human atherosclerosis.

**Supplementary Information:**

The online version contains supplementary material available at 10.1186/s13073-026-01613-1.

## Background

Immune cells play major roles in all stages of atherosclerosis [1]. While there is considerable information on αβ T cells in atherosclerosis, the contributions of γδ T cells in either murine or human atherosclerosis remain poorly understood [2]. γδ T cells develop and mature in the thymus. Subsequent to γδ TCR formation, the progenitors differentiate into subsets committed to produce either IL17 or IFNγ, referred to as γδ T17 or γδ T1 cells, respectively. Subsequently, both subtypes emigrate to the periphery. In peripheral tissues, γδ T cells show a marked preference for barrier tissues including skin and intestine indicating that they contribute to protecting the host from harmful exogenous antigens [3, 4]. While there is considerable information in mice regarding γδ T cell subtypes, there are controversial views on their human counterparts [5]. Moreover, differences in tissue distribution of γδ T cells are striking: For example, intestinal intraepithelial γδ T cells may constitute 40% of all T cells in both mice and humans but estimates of their frequency in the circulation range from 1 to 5% of all blood T cells [6, 7]. Of note, in contrast to atherosclerosis, their role in diseases as diverse as cancer, infectious diseases and autoimmune diseases is beginning to be understood at molecular levels [6, 8, 9]. The proinflammatory features of IL17-producing γδ T17 cells have been reported to promote disease progression in autoimmune diseases including inflammatory bowel disease, rheumatoid arthritis, and psoriasis [8]. Moreover, human γδ T cells with cytotoxic effector functions (cytolytic γδ T cells) have been shown to exhibit potent antitumor effects, prompting the development of multiple immunotherapeutic strategies targeting γδ T cells for the treatment of human cancers [9–12]. Thus, understanding the roles of γδ T17, γδ T1 cells, and cytolytic γδ T cells in atherosclerosis in both mice versus humans is important.

Unlike conventional αβ T cells, γδ T cells are viewed as innate-like T cells because they recognize and are activated by a diverse range of antigens such as phosphoantigens, endogenous and exogenous lipids, and multiple stress-induced molecules [4, 6]. Furthermore, γδ T cells also express toll-like and natural killer (NK) receptors adding to their ability to act as innate immune cells as first lines of defense towards exogenous and endogenous danger signals [6]. However, recent studies have also identified a series of traits related to adaptive immunity [6]. Unlike antigen recognition by αβ T cells which use MHC-I/II molecules presented by antigen-presenting cells (APCs), γδ T cells bind to antigens that are presented on CD1 molecules on both prototypic APCs and non-APCs, such as tumor cells and epithelial cells [13, 14]. This data indicates their considerable elasticity to adopt to exogenous antigens but their involvement in endogenous antigens remains to be better understood. The antigen recognition profile and the specific clonal expansion of γδ T cells reported in human cytomegalovirus infection and cancer revealed that γδ T cells may contribute via their TCR to recognize exogenous harmful antigens implying a potentially important adaptive immunity trait [15, 16]. Of note, recent evidence demonstrates their participation in intestinal nutrient sensing of ingested proteins, carbohydrates and lipids indicating their enormous adaptation capabilities to quickly respond to and control changes in environmental nutrients [17]. These studies are significant as they reveal that γδ T cells not only regulate responses towards exogenous microorganisms but also contribute to the regulation of nutrient metabolism. Yet, whether they participate in autoimmune reactions to self remains an important unanswered question.

Previous studies have reported conflicting roles of murine γδ T cells in atherosclerosis [18–20]. Deficiency of γδ T cells in *Apoe*^−/−^*TCRδ*^−/−^ mice resulted in reduced lipid accumulation in early atherosclerotic lesions under both chow-diet and high-fat diet (HFD) conditions [18]. Consistent with this observation, *Ldlr*^−/−^/*Il23r*^−/−^ mice exhibited smaller atherosclerotic lesions under a HFD diet suggesting a pro-atherogenic effect for γδ T cells [19]. On the other hand, no difference in atherosclerotic lesion formation was observed between *Apoe*^−/−^ mice and *Apoe*^−/−^/*TCRδ*^−/−^ mice after 10 weeks HFD feeding [20]. The role of γδ T cells in human atherosclerosis is even less defined. However, a functional dichotomy of γδ T cells has been observed in cancers between mice and humans, where mouse γδ T17 cells increase at tumor sites were they promote tumor growth via IL17, while human γδ T cells are intrinsically biased towards cytotoxic effector functions to exert protective effects [9, 21, 22]. These important species differences between mice and human pose significant challenges for extrapolating findings from mice to humans in multiple clinically important diseases and in atherosclerosis in particular.

We recently reported marked clonally expanded αβ T cell subtypes, i.e. CD4^+^, CD8^+^ and CD4^+^ T regulatory (T_reg_) cells in atherosclerotic plaques and ATLOs [23]. These studies provided evidence that atherosclerosis may have a significant T cell-dependent autoimmune component due to dysfunction of peripheral tolerance but whether γδ T are clonally expanded and whether tolerance dysfunction might be involved remains an important unanswered question. Moreover, significantly, γδ T cells have recently been shown to affect the formation of germinal centers in lymph nodes thereby acquiring an impactful role in the maturation of B cell receptors leading to high-affinity antibody responses [24]. These studies caught our attention as to the potential roles of γδ T cells in bridging innate and adaptive immunity in atherosclerosis. In addition, γδ T cells were observed to increase in plaques versus lymph nodes indicating that they participate in plaque immune responses [23]. However, there is no information on gene expression profiles or clonal expansion of γδ T cells in atherosclerosis. Moreover, few studies have investigated γδ T cells across different mouse models, ages and peripheral tissues and to examine whether data in atherosclerosis-prone mice are translatable to the human disease. Here, we combined scRNA-seq and scTCR-seq, utilized spatial transcriptomics to delineate their anatomical location within the diseased arterial wall, and employed multi-dataset scRNA-seq integration to compare γδ T cell phenotypes in murine versus human atherosclerosis. Our data revealed that the majority of diseased artery γδ T cells locate to ATLOs, that a clonally expanded γδ eduT17 subtype emerges in mouse plaques and ATLOs where they undergo disease-specific education but that the mouse data may not immediately be translatable to human atherosclerosis.

## Methods

### Mouse spatial transcriptomic sequencing and analysis

Aorta and renal lymph nodes (RLNs) from 78 weeks old *Apoe*^−/−^ mice were dissected as previously described [23]. Briefly, mice were housed under specific pathogen-free animal facilities of Munich University with a 12-h light/dark cycle, in an air-conditioned room (23 °C and 60% relative humidity). All mice were maintained on a standard rodent chow. Mice were euthanized by ketamine hydrochloride and xylazine hydrochloride. Blood was collected by cardiac puncture. Perfusion was performed from the left ventricle with 10 ml 5 mM EDTA buffer, 20 ml PBS and 20 ml FACS buffer. Animal procedures were conducted according to guidelines of the local Animal Use and Care Committees, and the National Animal Welfare Laws in compliance with European Community specifications on the use of laboratory animals. Aorta and RLN were collected and processed following the 10x Genomics Visium HD Fixed Frozen Tissue Preparation Handbook (CG000764, Rev A). Briefly, 10 μm tissue sections were prepared using a cryostat (Leica) and mounted on Superfrost Plus Slides (Thermofisher). Slides were stored at -80 °C until further processing. All subsequent steps, including tissue processing, imaging, and library construction, were performed according to the 10x Genomics Visium HD Spatial Gene Expression Reagent Kits User Guide (CG000685, Rev B). Tissue sections were stained with H&E and imaged with an Aperio slide scanner (Leica). Following imaging, sections underwent decrosslinking, destaining and overnight probe hybridization with the 10x Visium HD probe set. Hybridized probes were released from the tissue, ligated to spatially barcoded oligonucleotides on the Visium HD gene expression slide, and amplified to construct sequencing library. Library was sequenced on a NovaSeq-X Plus platform according to the manufacturer’s instructions. Raw sequencing reads were aligned to probe sequences and mapped to the corresponding CytAssist image using Spaceranger (version 3.1.2). The resulting output data, including 2 × 2 μm filtered barcode matrix, parquet tissue position matrix and high-resolution H&E image, were used for downstream nucleus segmentation analysis. Nucleus segmentation was performed using a custom python script based on StarDist and provided by 10x Genomics (https://www.10xgenomics.com/cn/analysis-guides/segmentation-visium-hd) [25]. The resulting gene-by-binned barcode data was analyzed in R (version 4.4.3) for cell annotation and visualization. Cell deconvolution was conducted using Robust Cell Type Decomposition (RCTD, spacexr package, version 2.2.1), leveraging an integrated scRNA-seq datasets from atherosclerotic tissues as the reference for cell type annotation. The analysis was performed in doublet mode, and spots classified as ‘reject’ were filtered out, retaining only the cell type with the higher predicted weight between predicted first and second cell type. Annotated cell types were displayed on the H&E image by using SpatialDimPlot function in Seurat (version 4.3.0).

To account for potential transcript dropouts associated with nucleus-based segmentation, spatial transcriptomic data were also analyzed using a fixed-size binning approach. Specifically, 8-µm binned data were adopted in downstream analyses to assess the spatial distribution and expression levels of *Trdc* across different tissue locations.

### Immunofluorescence staining

Mouse aortas and RLNs were prepared and embedded in Tissue-Tek (Sakura Finetek), frozen in chilled isopentane over dry ice, and stored at − 80 °C until cryosection. Serial frozen tissue Sect. (10 μm thick) were prepared. Immunofluorescent staining was performed using the following antibodies: anti-mouse CD3 (14-0032-85, Invitrogen, 1:100) and anti-mouse γδ TCR (118101, BioLegend; 1:100). Secondary antibodies were applied as previously described [23]. For negative controls, staining was performed without primary antibodies. Stained sections were analyzed using a Leica SP8 3X confocal laser scanning microscope (Mannheim, Germany).

### Mouse scRNA-seq data collection and integration

For the integration analysis of mouse scRNA-seq data, we downloaded publicly available scRNA-seq datasets from Gene expression omnibus database. The datasets included in our current analysis are (i) SMC-lineage tracing mice with *Ldlr*^−/−^ or *Apoe*^−/−^ genetic background were used as the mouse model. Aortas from these mice fed a HFD were collected for scRNA-seq analysis (GSE155513) [26]; (ii) Aortas of *Ldlr*^−/−^ mice fed with different weeks of HFD were used for analysis (GSE97310) [27]; (iii) Aortic CD45^+^ cells extracted from C57BL/6 and early atherosclerotic *Ldlr*^−/−^ mice were used for analysis (GSE154817) [28]; (iv) Aortic cells extracted from C57BL/6 mice and atherosclerotic *Ldlr*^*−/−*^*/Apob*^*100/100*^ mice fed different weeks of HFD were collected for scRNA-seq analysis (GSE205930) [29]; (v) T cells from young and aged C57BL/6 mice and T cells from young and aged atherosclerotic AAV-PCSK9 mice were collected for analysis (GSE210719) [30]; (vi) CD45^+^ cells sorted from atherosclerotic AAV-PCSK9 mice aortas, which were used to study Treg cells in atherosclerosis regression, were leveraged for scRNA-seq analysis (GSE141038) [31]; (vii) Sorted CD45^+^ cells from aortas of atherosclerotic *Apoe*^−/−^ mice fed normal diet or HFD were collected for analysis (GSE190220) [32]; (viii) Aged C57BL/6 and *Apoe*^−/−^ mice fed normal diet used in our previous study were also included for current integration analysis. Samples in our previous study included peripheral blood from C57BL/6 and *Apoe*^−/−^ mice, aorta draining lymph nodes from both mouse strains, artery tertiary lymph nodes and aortic plaques from *Apoe*^−/−^ mice. CD45^+^ cells from these samples were extracted from scRNA-seq analysis (10.6084/m9.figshare.21900735. v2) [23]. All the above data were loaded into R and integrated using the Seurat package (version 4.3.0). Low quality cells and cell doublets were filtered out based on the criteria of the number of genes detected per cell and the average expression level of mitochondrial genes in each study. Otherwise, cells were retained by limiting the number of genes detected in each cell to more than 200 and less than 4000, and the average expression level of mitochondrial genes was less than 8%. After quality control, genes were normalized across cells and the top 2000 highly variable genes were identified in each sample. These highly variable genes were scaled and further used for principal component analysis (PCA). Anchors were identified by anchor-based reciprocal principal component analysis (RPCA) in Seurat and representative samples from each dataset were selected as references for integration. The top 10 significant principal components (PCs) were used to construct the shared nearest-neighbor (SNN) graph and the top 10 significant PCs were used for uniform manifold approximation and projection (UMAP) visualization. Cell clusters were defined by setting the resolution to 1 for total cell analysis. The major cell types were defined for further sub-clustering by using cell type specific markers. The integration local inverse Simpson’s indices (iLISI) score quantifies the diversity of batch labels within the local neighborhood of each cell, serving as a metric to evaluate the effectiveness of batch effect correction in scRNA-seq data integration. A higher iLISI score indicates more successful batch effect removal [33]. This score is calculated using the ‘compute_lisi’ function from the lisi package (version 1.0).

### Mouse γδ T cell integration and sub-clustering

For further analyses of γδ T cells, we first extracted all total T cells from the above integrated dataset. We then re-clustered these T cells using RPCA. γδ T cell clusters are recognized by expression of *Trdc* and *Tcrg-C1*, but not *Cd4* or *Cd8a*. To more deeply clarify the features of γδ T cells in atherosclerosis, we integrated the identified γδ T cells from integrated dataset with FACS-sorted γδ T cells from thymus, peripheral lymph nodes and spleen of C57BL/6 mice (GSE179422) [34]. Cells were integrated by RPCA. The top 35 significant PCs were used to construct the SNN graph and the top 35 significant PCs were used for UMAP visualization. Cell clusters were defined by setting the resolution to 0.9. γδ T cell clusters were recognized by using the reported specific gene markers [35, 36]. Cell cycle status of γδ T cells were determined using the CellCycleScoring function in Seurat, which assigns each cell a cell cycle score based on the gene expression of G2/M and S phase markers in each cell.

To compare the gene expression profiles of γδ T17 cells in diseased arteries and ATLOs with those from homeostatic skin tissues (GSE123400) [35]. we extracted γδ T17 cells from our integrated γδ T cell dataset and combined them with publicly available homeostatic skin γδ T17 cells, following the same integration analysis pipeline described for γδ T cell analyses.

### Human scRNA-seq data collection and integration

The publicly available human scRNA-seq data associated with atherosclerotic plaques under various conditions were download from Gene expression omnibus or Zenodo for integration. These datasets include: (i) symptomatic or asymptomatic carotid plaques patients undergoing carotid endarterectomy. CD45^+^ leukocytes were collected for scRNA-seq analysis (GSE224273) [37]; (ii) Plaque specimens were obtained from patients undergoing carotid endarterectomy. Patients were divided into symptomatic and asymptomatic based on whether they suffered stroke or a transient ischemic attack. All cell types in the patient plaque specimens were used for scRNA-seq (GSE253904) [38]; (iii) Calcified atherosclerotic core and patient-matched proximal adjacent carotid arteries from patients undergoing carotid endarterectomy were collected. All cell types in the samples were used for scRNA-seq analysis (GSE159677) [39]; (iv) Carotid plaques and femoral plaques were collected from patients undergoing endarterectomy. Total leukocytes were obtained for scRNA-seq analysis (GSE234077) [40]; (v) Cardiac aorta, coronary artery and pulmonary arteries obtained from patients who had undergone heart transplantation. Samples in this study were absent of calcification or stenosis verified via preoperative computed tomography or coronary angiography. All cell types were used for scRNA-seq analysis. The scRNA-seq data were download from zenodo (https://zenodo.org/records/6032099) [41]; (vi) to fully understand the profiles of γδ T cells, we also included the human peripheral blood samples from young, old and diseased individuals for integration analysis (GSE157007) [42]. All data were loaded into R and processed by Seurat (version 4.3.0). Low-quality cells and cell doublets in each sample were filtered out by limiting the number of genes detected in each cell to more than 200 and less than 4000, and the average expression level of mitochondrial genes was less than 10. The data were then normalized and log transformed to remove the confounding of different sequence depths. 2000 highly variable genes among cells were identified in each sample, which were scaled and further used for PCA analysis. Anchors were identified by RPCA method in Seurat and representative samples from each dataset were selected as references for integration. The top 20 significant PCs were used to construct the SNN graph and the top 20 significant PCs were used for UMAP visualization. Cell clusters were defined by setting the resolution to 1 for total cell analysis. The major cell types were identified for further sub-clustering using cell type specific markers.

### Human γδ T cell integration and sub-clustering

T cells were extracted and sub-clustered using the procedures described above. The top 30 significant PCs were used to construct the SNN graph and the same top 30 significant PCs were employed for UMAP visualization. Cell clusters were defined by setting the resolution to 1. γδ T cells were grouped into a single cluster and a large number of *TRDC*^+^ cells scattered among other clusters. To completely separate γδ T cells, we took a strategy to extract the cluster defined as γδ T cells (*CD4*^−^*CD8A*^−^*TRDC*^+^*TRGV9*^hi^*TRDV2*^hi^) and further included T cells expressing *TRDC*, which is exclusively expressed in γδ T cells. To elucidate the differences of γδ T cells between human atherosclerotic plaques and other tissues, we further incorporated a public FACS-sorted human γδ T cell scRNA-seq data, which originate from blood, spleens, lungs, LLNs (lung-associated lymph nodes or thoracic lymph nodes), jejuna, and MLNs (mesenteric lymph nodes) of adult donors [43]. The data were downloaded from https://cellxgene.cziscience.com/collections/ec691f5f-0aac-433c-8f78-e7f4b85a05e0. The ensembl gene IDs used in this data were converted to gene symbol by using biomaRt (version 2.54.1). The top 16 significant PCs were used to construct the SNN graph and the same top 20 significant PCs were used for UMAP visualization. Cell clusters were identified by setting the resolution to 0.7. γδ T cell clusters were identified using differentially expressed genes (DEGs) and reported cell markers [43]. Based on the gene expression profiles, γδ T cell clusters were further grouped into five major cell types. The percentage of γδ T in T cells were calculated as: number of γδ T cells in each sample ÷ number of total T cells in each sample × 100%. Samples with fewer than 20 total T cells were excluded from this analysis to avoid biases caused by inaccurate estimations. The average percentage of different γδ T cell clusters or cell types in different tissues or conditions were calculated as: number of γδ T cell clusters or cell types ÷ total number of γδ T cells in each tissue or condition × 100%. The cell percentage were displayed using bar plot or donut chart (‘PieDonut’ function in webr package, version 0.1.6). To assess the batch effect correction after integration, we employed the iLISI score as described in mouse study as well.

### Calculation the difference of cell percentages between asymptomatic and symptomatic patients

Following the sub-clustering analyses of T cells, we performed similar analyses for B cells, myeloid cells and non-immune cells. Cell subtypes were defined based on significant DEGs and reported cell markers. Low-quality cells and cell doublets were excluded from further cell percentage calculations. This cell type information was merged back into the metadata of the total integrated scRNA-seq data. The average percentage of each cell subtype was calculated as follows: the total number of one certain cell subtype in asymptomatic or symptomatic plaque samples divided by the total number of asymptomatic or symptomatic plaque samples × 100%. The difference between asymptomatic and symptomatic patients for a given cell subtype was calculated as: the percentage of a given cell subtype in asymptomatic patients minus the percentage of its counterpart in symptomatic patients. Differences in cell percentages were scaled for ease of display, and results were shown using the pheatmap package (version 1.0.12).

### Gene ontology (GO) enrichment analysis

Significant DEGs were used as the input genes. Pathways were enriched by using ‘enrichGO’ function in the clusterProfiler package (version 4.6.2). The enrichment results were first re-arranged by ‘circle_dat’ function in the GOplot package (version 1.0.2) to generate a zscore for each pathway. A positive zscore value indicates that a great number of genes associated with one specific pathway are upregulated in the dataset. Pathways associated with biological process were visualized using bubble plot. Pathways were ranked primarily by statistical significance (-log10(adjusted p value)) and secondarily by absolute z-score value. Top 10 significantly enriched pathways for each cluster were annotated in Figures.

### Gene set enrichment analysis (GSEA)

To assess functional differences between effector/cytolytic and Th17-like γδ T cells across different plaque stability states, DEGs identified by ‘FindMarkers’ function was subjected to GSEA analysis. Pathway enrichment was performed using the ‘GSEA’ function from the clusterProfiler package (version 4.6.2), and results were visualized with the ‘gseaNb’ function from the GseaVis package (version 0.0.5). Pathways with a higher absolute normalized enrichment score and an adjusted p value less than 0.05 were considered significantly enriched.

### Gene signature module score analysis

Gene signature module scores were calculated using the ‘AddModuleScore_UCell’ function from the UCell package (version 2.2.0). The anti-apoptotic gene signature included *Bcl2*, *Bcl2l1*, *Mcl1*, *Bcl2l12*, *Bcl2l13*, *Birc2*, *Birc3*, *Birc5*, *Birc6*, *Xiap*, *Bcl2a1a*, *Bcl2a1b*, *Bcl2a1c*, and *Bcl2a1d*. For metabolic program evaluation, curated marker genes representing distinct metabolic pathways are used as follows: Lipogenesis: *Srebf1*, *Srebf2*, *Acaca*, *Fasn*, *Acly*, *Scd1*, *Elovl6*, *Hmgcr*, *Hmgcs1*, *Sqle*, and *Fdps*; Fatty-acid oxidation: *Ppara*, *Acox1*, *Cpta1*, *Acadm*, *Pdk4*, *Acadvl*, *Hadha*, *Hadhb*, *Cpt2*, *Ehhadh*, and *Hmgcs2*; Lipid uptake: *Ldlr*, *Scarb1*, *Npc1*, *Npc2*, and *Cd36*; Glycolysis: *Hk1*, *Hk2*, *Pfkm*, *Pfkp*, *Aldoa*, *Tpi1*, *Gapdh*, *Pgk1*, *Pgam1*, *Eno1*, and *Pkm*; Oxidative phosphorylation: *Ndufs1*, *Ndufs2*, *Ndufv1*, *Sdha*, *Sdhb*, *Uqcrc1*, *Uqcrc2*, *Cox4i1*, *Cox5a*, *Atp5f1a*, *Atp5f1b*, and *Atp5mc1*; Lipid storage: *Gpam*, *Agpat2*, *Lpin1*, *Dgat1*, *Dgat2*, *Plin2*, and *Plin3*. In addition, in human data, NK gene signature scores were calculated using *NCAM1*, *NCR1*, *NCR3*, *FCGR3A*, *SPON2*, *FCER1G*, *TYROBP*, *KLRB1*, *KLRD1*, and *KLRC1*. Cytotoxic gene signature scores were computed based on *GZMB*, *GZMA*, *GZMH*, *PRF1*, *NKG7*, *GNLY*, *CCL5*, *CCL4*, *CCL4L2*, *CCL3*, *GZMK*, *CTSW*, *XCL1*, and *XCL2*.

### Cell-cell communication modeling

To explore the differences of cell-cell interactions between human γδ T cells and other immune and non-immune cells. CellphoneDB (version 5) was adopted to predict the significant interaction across different cell types in Python. The output interaction scores, means, and p values files were combined to define the significant interactions. The interactions with interaction score more than 0 and p values less than 0.05 were considered as significant interactions. P values less than 0.000001 were re-set to 0.000001 for better data visualization.

### TCR reconstruction and clonal expansion analyses of γδ T cells in mouse atherosclerosis

Given the different study purposes and technical limitations of the available dataset, we employed TRUST4 (version 1.1.5) to reconstruct the TCR from scRNA-seq data derived from 5’ 10x scRNA-seq library without V(D)J enrichment [44]. TRUST4 enables de novo assembly of the V, D, J genes, as well as the CDR3 region, followed by annotation through alignment to IMGT reference gene sequences. In our collected mouse atherosclerosis data, only two datasets -those from Tyrrell et al. [30] and our own group [23]- were generated using 5’ scRNA-seq data (Additional file 2: Table S1). For the Tyrrell et al. study, raw fastq files were retrieved from the GEO database. For the reconstruction of mouse TCR/BCR, we utilized the GRCm38_bcrtcr.fa, which provides the genomic coordinates and sequences of V/D/J/C genes, alongside the mouse_IMGT + C.fa file for contig annotation. The readFormat was configured as bc:0:15, r1:26:-1. The barcode whitelist was specified according to the sequencing kits employed in each study. The output barcode report file contains the cell type and TCR/BCR information of annotated cell, we filtered out non-γδ T cells and subsequently quantified the percentage of TRGV family and evaluated the diversity of the CDR3 within the γ chains. Bulk RNA-seq data from human carotid plaques, classified by symptomatic status (GSE198600) and plaque stability (GSE120521), were retrieved from GEO database. According, hg38_bcrtcr.fa and human_IMGT + C.fa were used to reconstruct human TCR/BCR repertoires in each sample. The resulting output report files were utilized for downstream analysis. Out-of-frame CDR3 sequences were excluded prior to further evaluation.

### Statistical analysis

Data were analyzed in R by using stats package (version 4.2.2). The data distribution was firstly tested by Shapiro-Wilk test (‘shapiro.test’ function in stats package). For data that followed a Gaussian distribution, a one-way analysis of variance (ANOVA) was used to perform statistical analysis among multiple groups (‘aov’ function in stats package). If datasets did not follow Gaussian distribution, the difference between two groups was compared by two-sided Wilcoxon rank-sum test (‘wilcox.test’ function in stats package) and difference between three or more groups was analyzed by non-parametric Kruskal–Wallis *H* test (‘kruskal.test’ function in stats package). Differences were considered significant at a two-tailed *P* value < 0.05.

## Results

### γδ T cells increase in ATLOs and atherosclerotic plaques during mouse atherosclerosis progression

**Fig. 1:**
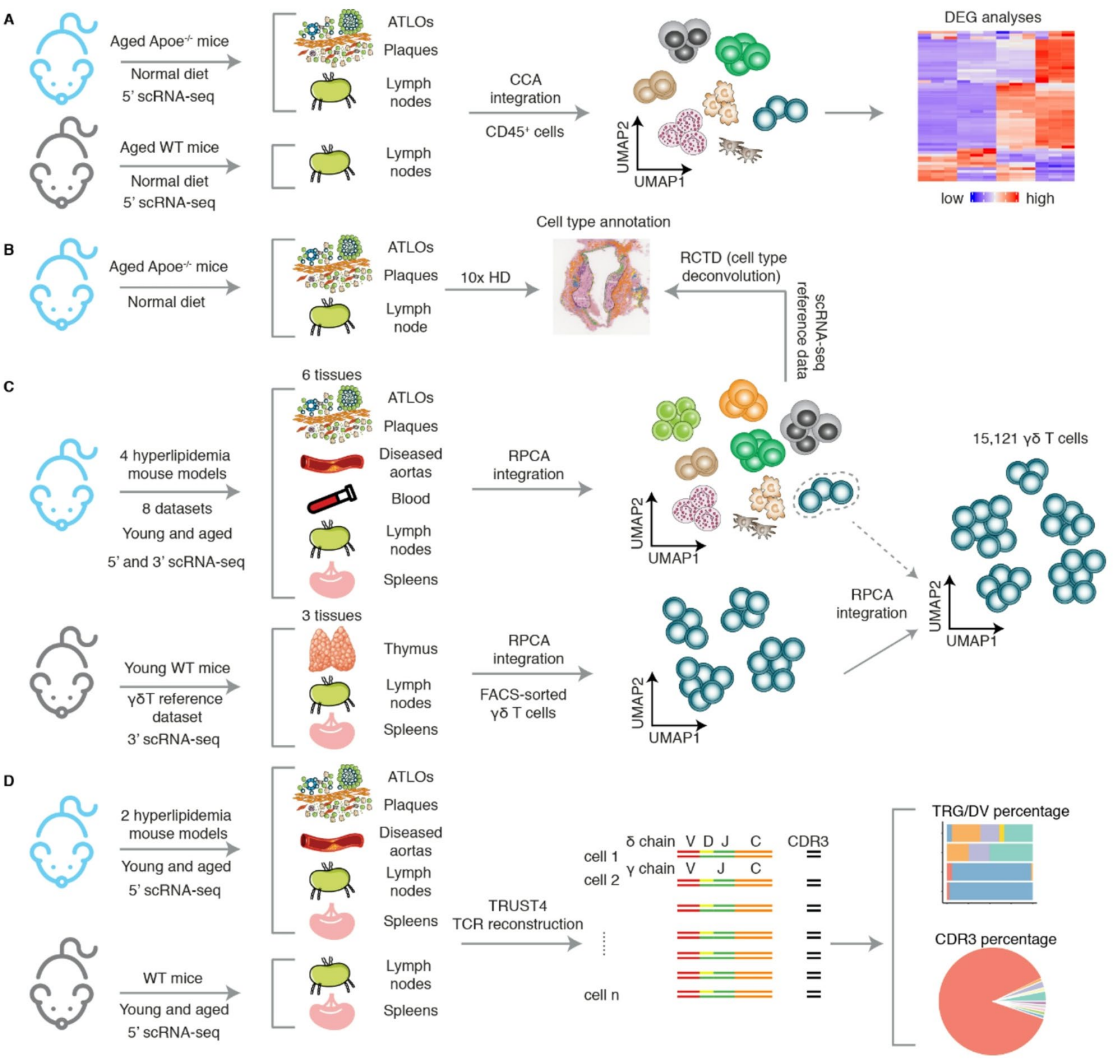
Three-pronged approach combining scRNA-seq/scTCR-seq, spatial transcriptomics and multi-sample scRNA-seq integration in atherosclerosis.** A.** 5’ scRNA-seq analyses reveal the diversity of γδ T cells in advanced atherosclerotic *Apoe*^−/−^ mice, with aorta-draining lymph nodes from aged and sex-matched WT mice serving as controls. CCA, Canonical correlation analysis. **B.** Spatial transcriptomic sequencing maps the distribution of γδ T cells and other cell types within atherosclerotic plaques, ATLOs and aorta-draining lymph nodes. RCTD, Robust cell type decomposition. **C.** Anchor-based RPCA integration of multi-tissue scRNA-seq datasets enables comprehensive analyses across different samples. RPCA, Reciprocal principal component analysis. **D.** TRUST4-based reconstruction of TCRs from 5’ scRNA-seq datasets associated with atherosclerosis to determine the TCR repertoire diversity in mouse atherosclerosis. CDR3, Complementarity-determining region 3

**Fig. 2:**
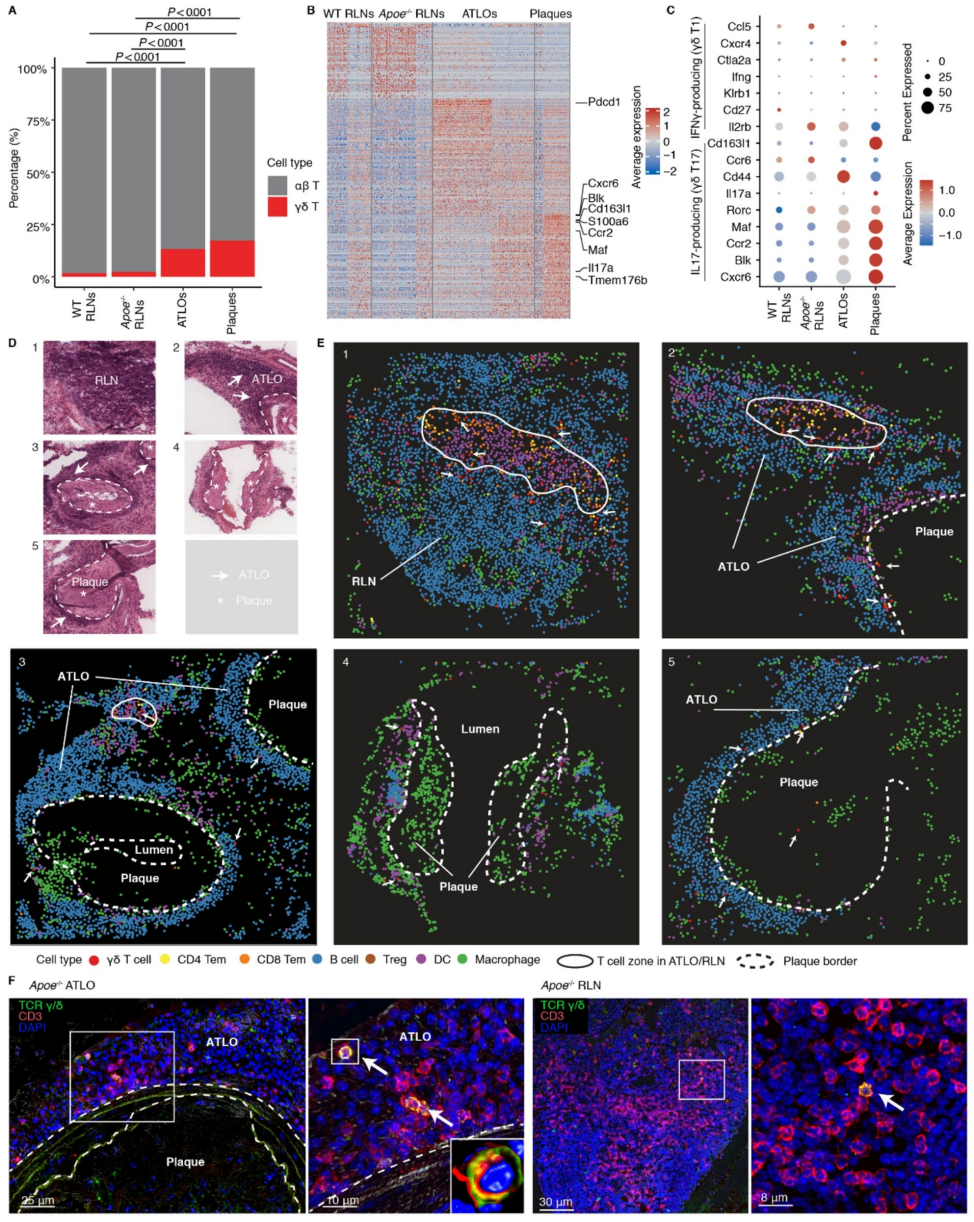
ScRNA-seq and spatial transcriptomic analyses reveal phenotypes and spatial distribution of γδ T cells in advanced atherosclerosis. **A **Percentage of αβ T cells and γδ T cells across various tissues in aged WT and *Apoe*^−/−^ mice. Pairwise comparisons between groups were performed using Chi-square tests. P values were adjusted for multiple comparisons using Bonferroni analysis. **B **Heatmap visualizes differentially expressed transcripts in various tissues of aged WT vs. *Apoe*^−/−^ mice. **C **Average gene expression levels of markers associated wtih IFNγ-producing and IL17-producing γδ T cell subtypes in different tissues of aged WT and *Apoe*^−/−^ mice. **D **H&E staining illustrates the histological structure of advanced atherosclerotic aorta and diseased aorta associated ATLO and RLN of aged *Apoe*^−/−^ mice. Representative images displaying different disease regions. Locations are numbered in the top left corner. Dashed lines represent plaque-media border. Arrow represents ATLO. Asterisk represent atherosclerotic plaque. **E **Representative images displaying the distribution of γδ T cells along with other immune cells across distinct tissue regions. Locations are numbered in the top left corner, corresponding to regions in panel D. Location 1 represents a RLN; Location 2, 3 represent atherosclerotic plaques and ATLOs, respectively; Location 4 represents an atherosclerotic plaque without adventitial immune cell aggregates; Location 5 represents an atherosclerotic plaque with an immature ATLO. Dashed lines represent plaque-media border. Solid lines represent border of T cell and B cell regions in RLN and ATLOs. Arrows represent γδ T cells. **F **Immunofluorescence staining illustrates the distribution of γδ T cells in various tissues from mice with advanced atherosclerosis. Representative images are shown for ATLO and RLN in aged *Apoe*^−/−^ mice. The white squares indicate the regions magnified in the adjacent zoomed-in panels. Arrows represent γδ T cells

To understand roles of γδ T cells in atherosclerosis, we employed a combined three-pronged approach using scRNA-seq/scTCR-seq, spatial transcriptomics and multi-dataset scRNA-seq integration (Fig. 1). This approach allowed us to identify γδ T cell subtypes and profile transcriptomes in distinct subtypes, and to examine their innate and potentially adaptive immune response characteristics. Moreover, we aimed at identifying their anatomical location in diseased arteries and evaluating whether mouse characteristics are translatable to human atherosclerosis. Specifically, we employed this combined approach across atherosclerotic plaques of wild-type (WT) and hyperlipidemic mouse models and human plaques, atherosclerosis-specific mouse ATLOs, and aorta-draining RLNs. Our analyses revealed a significant increase of γδ T cell percentages in atherosclerotic plaques and ATLOs in contrast to aorta-draining secondary lymphoid organs (SLOs) of WT and *Apoe*^−/−^ mice (Figs. 1A and 2A), suggesting a significant involvement of γδ T cells in the progression of atherosclerosis. Furthermore, we observed marked intra- and inter-tissue transcriptional heterogeneity among γδ T cells across plaques, ATLOs and SLOs (Fig. 2B). It is well established that γδ T cells commit into IFNγ-producing (γδ T1) or IL17-producing (γδ T17) cells in mice during intra-thymic maturation [4]. We therefore visualized highly expressed genes associated with γδ T1 and γδ T17 cells. Our data suggested that γδ T17 cells predominated in these tissues, as genes associated with γδ T17 cells were highly expressed, being particularly pronounced in plaques (Fig. 2B, C). In contrast, marker genes of γδ T1 cells were less abundant in these tissues, suggesting a specific role of γδ T17 cells in atherosclerosis.

To verify our findings and investigate the spatial distribution of γδ T cells in advanced atherosclerosis, we conducted spatial transcriptomic analyses using 10x Visium HD approach and applied a nucleus-based segmentation strategy (Figs. 1B and 2D). Our analyses revealed high numbers of detected genes per single immune and nonimmune cells across plaques, ATLOs, and RLNs (Additional file 1: Fig. S1A, B). The cell type distribution showed: fibroblasts were predominantly located in the adventitia, modulated smooth muscle cells were widespread in atherosclerotic lesions [26], and high numbers of T and B cells were observed in RLNs and ATLOs, confirming the quality of our cell annotation (Additional file 1: Fig. S1B). We further examined whether γδ T cells exhibited distinct spatial distribution preferences in advanced atherosclerosis. For example, within the 219 γδ T cells per single mouse aorta identified in spatial transcriptomic analysis, only 7 were found in atherosclerotic plaques, 62 in lymph nodes, and 121 in the diseased adventitia/ATLOs, with the remaining located in other regions, such as adipose tissue. These results demonstrated that γδ T cells predominantly localized in ATLOs, in close proximity to other immune cells, rather than within advanced atherosclerotic plaques (Fig. 2E). Notably, γδ T cells were enriched in the T cell zones and T-B cell border regions of RLNs and mature ATLOs (Fig. 2E). Additionally, γδ T cells showed close spatial associations with DCs, CD8^+^ T effector memory (em) cells, B cells, and T_reg_ cells in ATLOs, suggesting potential functional interactions. Immunofluorescent staining further confirmed the presence of γδ T cells in RLNs and ATLOs (Fig. 2F). Considering that the nucleus-based segmentation strategy may lead to transcript dropouts due to the exclusion of most cytoplasmic mRNAs, this approach may underestimate the detection of γδ T cells in spatial transcriptomics. To address this limitation, we performed an additional, fixed-size binning (8 μm bins, close to single-cell dimension) analysis to detect *Trdc*, a highly and specifically expressed marker of γδ T cells. This complementary approach substantially increased detection sensitivity and revealed a broader distribution of *Trdc*^+^ signals across the adventitia, ATLOs, and plaques (Additional file 1: Fig. S2). Notably, *Trdc*^+^ bins were more abundant than γδ T cells identified by nucleus-based segmentation at the same anatomical locations and consistently showed enrichment in the adventitia and ATLOs.

### Multi-dataset scRNA-seq integration uncovers functional diversity of γδ T cells in mouse atherosclerosis

To examine whether the characteristics of γδ T cells observed in advanced atherosclerosis of the *Apoe*^−/−^ mouse model are conserved across other mouse atherosclerosis models and disease stages, we incorporated multiple scRNA-seq datasets involving atherosclerotic plaques, atherosclerotic aortas, atherosclerosis-associated LNs, and peripheral blood derived from different atherosclerotic mouse models (*Apoe*^−/−^, *Ldlr*^−/−^, and AAV-PCSK9-induced atherosclerosis) across different ages (from 8 to 78 weeks) [23, 26–32] (Fig. 1C, Additional file 2: Table S1). We constructed a comprehensive landscape of γδ T cells in multiple murine atherosclerosis models using anchor-based RPCA. Of note, RPCA is superior in multiple sample integration compared to other integration approaches to correct batch effects among different samples of different models [33, 45]. Our data demonstrated robust sample integration, as evidenced by the sample distribution in the UMAP visualization and significantly elevated iLISI – i.e. a metric quantifying local inter-batch cell mixing with neighborhoods-for each dataset compared to corresponding datasets without batch effect correction (Additional file 1: Fig. S3A-C). Consistent with γδ T cells in advanced plaques, our analyses revealed a marked increase of γδ T cells across different mouse models and plaque stages (Additional file 1: Fig. S3D). In contrast to atherosclerotic plaques and ATLOs, γδ T cells were less abundant in peripheral blood, LNs, and spleens, suggesting preferential accumulation in atherosclerotic lesions. In addition, to more precisely define γδ T cell subtypes and their characteristics under different disease conditions, we further integrated FACS-sorted γδ T cells extracted from thymus, peripheral LNs and spleen of healthy C57BL/6 mice as reported [34] (Fig. 1C). This integration strategy enhances the detection of rare γδ T cell subtypes, improves the resolution of subtype classification, and facilitates robust cross-dataset comparisons.

**Fig. 3:**
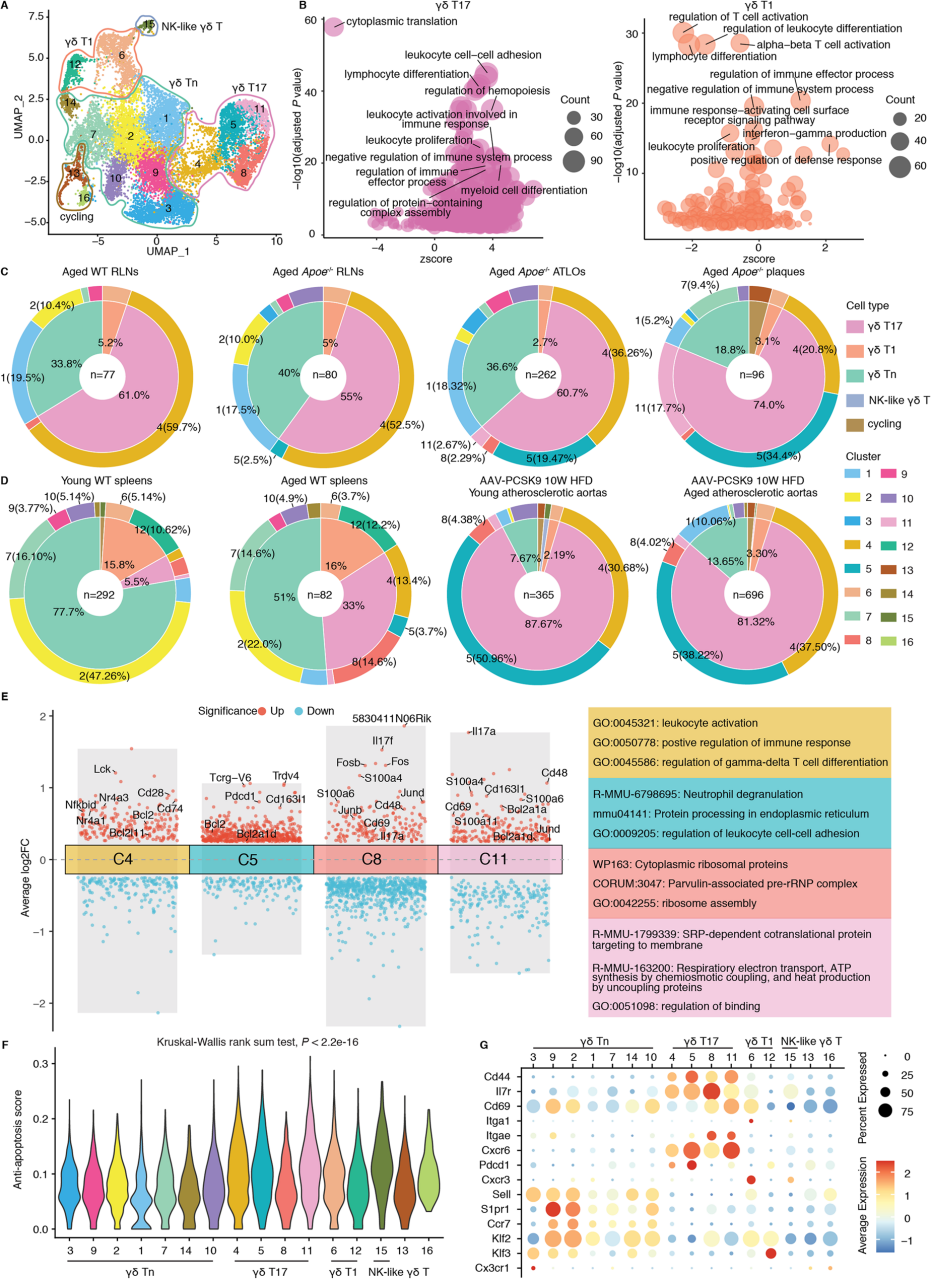
Multi-sample scRNA-seq integration analyses indicate numerical changes and potential functionally relevant impacts of γδ T cells in atherosclerosis. **A **UMAP plot illustrates the distribution and diversity of mouse γδ T cell subtypes in integrated datasets. **B **Bubble plots depict GO pathway enrichment analysis for γδ T17 and γδ T1 cell subsets. Dot size represents the count of genes enriched in each pathway. **C **Cell percentage of each cell subtype type or cluster in different tissues of aged WT and *Apoe*^*−/−*^ mice fed with a normal chow-diet. The inner circle represents different γδ T cell subtypes. The outer circle represents the cell clusters. **D **Cell percentage of each cell type or cluster in young and aged WT mice and HFD-treated AAV-PCSK9-induced atherosclerotic mice are shown. The inner circle represents different γδ T cell subtypes. The outer circle represents cell clusters. **E **Differential gene expression analysis showing up- and down-regulated genes across the γδ T17 cell subclusters (Left). Pathway enrichment analysis of γδ T17 cell subclusters (Right). The top three pathways for each cluster are shown. **F **Anti-apoptotic module score of each γδ T cell cluster. Kruskal-Wallis rank sum test was used to compared the differences among different clusters and γδ T cell subtypes. **G **Average gene expression levels of T cell tissue-resident-associated signature genes among different clusters and γδ T cell subtypes

All γδ T cells were separated into 16 clusters (Fig. 3A). High expression levels of *Sell*, *S1pr1*, *Ccr7*, *Lef1*, *Tcf7* and ribosomal related genes in cluster 1 (C1), C2, C3, C7, C9, C10, and C14 indicates a naïve subtype of γδ T cells [46], thus we designated these clusters as naïve γδ T cells (γδ Tn cells) (Additional file 1: Fig. S4A). In particular, C3, which exhibited exclusively high expression of *Ccr9*, *Sox13*, *Etv5* and *Gzma* [47], was almost exclusively enriched in the thymus (Additional file 1: Fig. S5A), indicating progenitor γδ T cells. C4, C5, C8, and C11 displayed higher expression of *Tmem176a*, *Cd163l1* (*Scart1*), *S100a4*, *S100a6*, *Cxcr6*, *Rorc*, *Rora*, *Blk*, *Il17a*, *Il17f*, *Ccr2*, *Ccr6*, and *5830411N06Rik* (*Scart2*), suggesting IL17-producing γδ T cells (γδ T17 cells) [35]. Of note, C4, C5 and C11 additionally exhibited higher levels of *Cd163l1* and *Tcrg-V6*, suggesting Vγ6^+^ γδ T17 cells, while, C8 showed specific expression of *5830411N06Rik*, indicating Vγ4^+^ γδ T17 cells. C6 and C12 both expressed *Ifng* along with different levels of *Klrb1c*, *Tbx21*, *Ly6c2*, *Cd160*, *Ccl5*, *Il2rb*, *Klrd1*, *Klrc1*, *Ctla2a*, and *Cxcr4*, all of which were markers for γδ T1 cells [36, 47]. C15 exhibited relatively high levels of γδ T1 cell markers and, in addition, along with specific expression of *Zbtb16* and *Il4*, distinguishing it as natural killer-like γδ T cells (NK-like γδ T cells) [6, 36, 48]. C13 and C16 showed higher levels of cell cycle related genes, further, cell cycle score analysis also confirmed the cycling cells of these two clusters (Additional file 1: Fig. S4B). To infer the potential functions of different γδ T cell subtypes, we performed pathway enrichment analysis using significant DEGs for each γδ T cell subtypes. γδ T17 cells were extensively involved in regulating cell adhesion, immune cells activation, differentiation and proliferation (Fig. 3B, Additional file 2: Table S2). Whereas another major subset of effector γδ T cells, γδ T1, may influence other cells through direct cell killing and immune regulation, as they are primarily implicated in pathways associated with immune effector processes, interferon-gamma production, cell killing and cytotoxicity (Fig. 3B, Additional file 2: Table S3). NK-like γδ T cells showed pathway enrichment in immune cell activation, differentiation, proliferation, migration and cell killing (Additional file 1: Fig. S4C, Additional file 2: Table S4).

### γδ T17 cells are the major γδ T cell subtype in mouse plaques derived from diverse hyperlipidemic mouse models

We assessed the composition of γδ T cell subtypes across various tissues and disease conditions (Fig. 3C, D, Additional file 1: Fig. S5). Among all analyzed samples, γδ Tn, γδ T1, and γδ T17 were the major subtypes, while NK-like and cycling γδ T cells were rare distributed. In young WT mice, γδ Tn cells were the predominant γδ T cell subset in the thymus, peripheral LNs and spleens, with γδ T1 and γδ T17 cells being the other two major populations (Fig. 3D, Additional file 1: Fig. S5A). Next, we evaluated the γδ T cell subtype compositions across different tissues in our mouse model of advanced atherosclerosis mice of aged WT and *Apoe*^*−/−*^ mice on normal chow-diet. This data showed that the frequency of γδ T cell subtypes was identical across WT RLNs versus *Apoe*^*−/−*^ RLNs indicating that hyperlipidemia and/or atherosclerosis did not affect LN γδ cells during aging (Fig. 3C). γδ T17 cells constituted the majority of γδ T cells in these tissues. In contrast, increases of γδ T17 in atherosclerotic plaques were observed (Fig. 3C). Additionally, analysis of AAV-PCSK9-induced atherosclerotic mice and their corresponding WT controls revealed a marked age-associated increase in splenic γδ T17 cells (Fig. 3D). Specifically, the proportion of splenic γδ T17 cells increased from approximately 5.5% in young WT mice to 33% in aged WT mice, suggesting that an aging-associated phenomenon leads to enrichment of γδ T17 cells in the spleen (Fig. 3D). However, atherosclerotic disease was associated with a strong influence on γδ T17 cells. In diseased aortas, γδ T17 cells became the predominant γδ T cell subset, accounting for more than 80% of all γδ T cells even in young AAV-PCSK9-induced atherosclerosis, however, with no significant difference observed between young and aged atherosclerotic mice (Fig. 3D). This indicated that the inflammatory and lipid-rich tissue-specific microenvironment of murine atherosclerosis further drives γδ T17 cell enrichment. Furthermore, γδ T17 cell enrichment observed in a female-only dataset (AAV-PCSK9-induced atherosclerotic mice; Fig. 3D) was comparable to that detected in male-only atherosclerosis datasets (aged *Apoe*^−/−^ mice; Fig. 3C), suggesting that sex is not a primary determinant of γδ T17 cell enrichment. Notably, in contrast to γδ T17 cells, γδ T1 cells represented only a minority of γδ T cells in atherosclerotic arteries (Fig. 3C, D, Additional file 1: Fig. S5). The profile of higher percentages of γδ T17 cells in atherosclerotic plaques was also found in HFD-fed *Apoe*^−/−^ and *Ldlr*^−/−^ mice (Additional file 1: Fig. S5B-E). All of these findings pointed to a pro-atherogenic role of γδ T17 cells, which is consistent with observation that the reduction of γδ T17 cells in *Ldlr*^−/−^*Il23r*^−/−^ mice exhibited reduced atherosclerotic lesion formation compared to the *Ldlr*^−/−^ control mice [19].

### ATLOs and atherosclerotic plaques shape distinct gene expression profiles of γδ T17 cells revealing atherosclerosis-specific education

The increase of γδ T17 cells and their potential pro-atherogenic role prompted us to further explore the γδ T17 cells in atherosclerosis. Differential gene expression analysis among the four γδ T17 subclusters indicated distinct functional states. Cluster C4 exhibited elevated expression of activation-associated genes, including *Cd28*, *Cd74*, *Lck*, *Nr4a3*, *Nr4a1*, and *Nfkbia*, but lacked expression of prototypical effector cytokines (*Il17a* and *Il17f*), suggesting a pre-activated state of Vγ6^+^ γδ T17 cells (Fig. 3E, Additional file 1: Fig. S4A). In contrast, C5 displayed elevated expression levels of the exhaustion marker *Pdcd1*, while effector cytokine genes and other exhaustion hallmark marker genes were less expressed in C5. We therefore called these cells as a potential PD-1-associated hypofunctional Vγ6^+^ γδ T17 cells. Notably, the C8 and C11 clusters showed increased expression of effector-associated genes (*Il17a*, *S100a4*, *S100a6*, *Jund*, *Cd48* and *Cd69*), consistent with activated effector Vγ4^+^ and Vγ6^+^ γδ T17 cells, respectively. Pathway enrichment analysis further supported these distinctions, with C4 and C5 Vγ6^+^ γδ T17 cells enriched in leukocyte activation, cell adhesion, and other immune responses, whereas the effector states of C8 and C11 γδ T17 cells were preferentially associated with protein synthesis-related and cellular metabolism-related pathways (Fig. 3E).

We next examined the tissue distribution of these γδ T17 subclusters to assess whether local microenvironments may shape their functional states. In contrast to SLOs, PD-1-associated hypofunctional γδ T cells (C5) were preferentially enriched in atherosclerotic aortas/plaques and ATLOs, indicating that these disease-associated tissue niches may drive γδ T cell towards a hypofunctional phenotype in a site-specific manner (Fig. 3C, D). Additionally, the predominance of a hypofunctional γδ T-cell phenotype induced by the atherosclerotic plaque microenvironment was conserved across different mouse models of atherosclerosis (Additional file 1: Fig. S5).

In addition to functional state changes, we observed marked differences in survival-regulating transcripts among γδ T17 subsets. Vγ6^+^ γδ T17 (C4, C5 and C11) exhibited an extensive expression of anti-apoptotic genes (including *Bcl2*, *Bcl2a1d*, *Bcl2l11*, and *Bcl2a1a*) compared to Vγ4^+^ γδ T17 cells (C8) (Fig. 3E). Consistently, anti-apoptotic scoring revealed significantly higher scores in Vγ6^+^ γδ T17 cells relative to γδ Tn and γδ T1 cells (Fig. 3F). Moreover, Vγ4^+^ and Vγ6^+^ γδ T17 cells from atherosclerotic plaques exhibited elevated anti-apoptotic scores compared with their splenic counterparts in WT mice, supporting the interpretation that the plaque microenvironment enhances γδ T cells survival capacity, but more pronounced in Vγ6^+^ γδ T17 cells (Additional file 1: Fig. S6A). This feature was conserved across advanced disease models, as comparable anti-apoptotic scores were observed in plaques and ATLOs from aged *Apoe*^*−/−*^ mice and AAV-PCSK9-induced atherosclerotic mice (Additional file 1: Fig. S6A). Although the anti-apoptotic profile of Vγ6^+^ γδ T17 cells is well-established under homeostatic conditions, such as in skin tissues [35], Vγ6^+^ γδ T17 cells within atherosclerotic plaques and ATLOs revealed a distinct anti-apoptotic signature, with upregulated *Bcl2*, *Mcl1* and BIRC family genes compared with their homeostatic skin-resident counterparts (Additional file 1: Fig. S6B).

Beyond survival, the tissue-resident properties of γδ T cells also contribute to their peripheral immunosurveillance capacity. We previously reported the tissue-resident feature of conventional αβ T cells in atherosclerosis [23], but the tissue residency of γδ T cells in atherosclerosis remained unknown. Here, we found that γδ T17 cells, compared with other γδ T cell subtypes, showed pronounced expression of tissue-resident-associated markers (*Cd44*, *Il7r*, *Cd69*, *Itgae*, *Cxcr6*, *Pdcd1*) and lower levels of tissue egress-associated genes (*Sell*, *S1pr1*, *Ccr7*, *Klf2*, *Klf3*) [49] (Fig. 3G). More importantly, these tissue-resident features were more pronounced in plaques and ATLOs than SLOs in both Vγ4^+^ and Vγ6^+^ γδ T17 cells (Additional file 1: Fig. S6C). Given that Vγ6^+^ γδ T17 cells form the predominate in atherosclerotic plaques and ATLOs, we investigated their tissue-resident properties in diseased arteries compared to homeostatic skin. Our data revealed consistent expression of tissue-resident marker genes in Vγ6^+^ γδ T17 cells across all these conditions (Additional file 1: Fig. S6D). In addition, the exhaustion marker *Pdcd1* exhibited higher expression levels in Vγ6^+^ γδ T17 cells compared to Vγ4^+^ γδ T17 cells (Additional file 1: Fig. S6B). The combination of apparent enhanced anti-apoptotic capacity, tissue residency, and hypofunction-like features in plaques and ATLOs indicates disease-specific adaptation.

Metabolic reprogramming are critical determinants for immune cell functions in atherosclerosis, such as T cells and macrophages [50, 51]. While metabolic regulation in αβ T cells has been extensively characterized, the metabolic features of γδ T cells, particularly in the context of atherosclerosis, remain poorly understood [52]. To address this knowledge gap, we performed a consolidated functional analysis of lipid and energy-related metabolic pathways in γδ T cells across two independent mouse models of atherosclerosis, focusing on curated gene sets representing fatty-acid uptake and storage, fatty-acid oxidation, lipid synthesis, glycolysis and oxidative phosphorylation.

Across both models, γδ T17 cells within atherosclerotic plaques revealed a consistent metabolic reprogramming compared with their counterparts in secondary lymphoid organs (Additional file 1: Fig. S6E, F). Specifically, plaque-resident γδ T17 cells showed markedly increased signatures of cholesterol uptake, lipid storage, and glycolysis, accompanied by relatively basal mitochondrial oxidative metabolism. Notably, these metabolic alterations were largely shared between Vγ4⁺ and Vγ6⁺ γδ T17 cells, with only minimal differences observed between the two subsets within atherosclerotic plaques (Additional file 1: Fig. S6G). These findings indicate that atherosclerotic plaques and ATLO microenvironments impose a distinct metabolic program on γδ T17 cells, characterized by enhanced lipid uptake and storage coupled with elevated glycolysis. Collectively, these findings demonstrate that the microenvironments of atherosclerotic plaques and ATLOs may educate γδ T17 cells, predominantly Vγ6⁺ γδ T17 cells, and to a lesser extent Vγ4⁺ γδ T17 cells, into distinct phenotypes, which we term educated γδ T17 cells (γδ eduT17).

### TCR clonal expansion of γδ T cells in mouse atherosclerosis

**Fig. 4:**
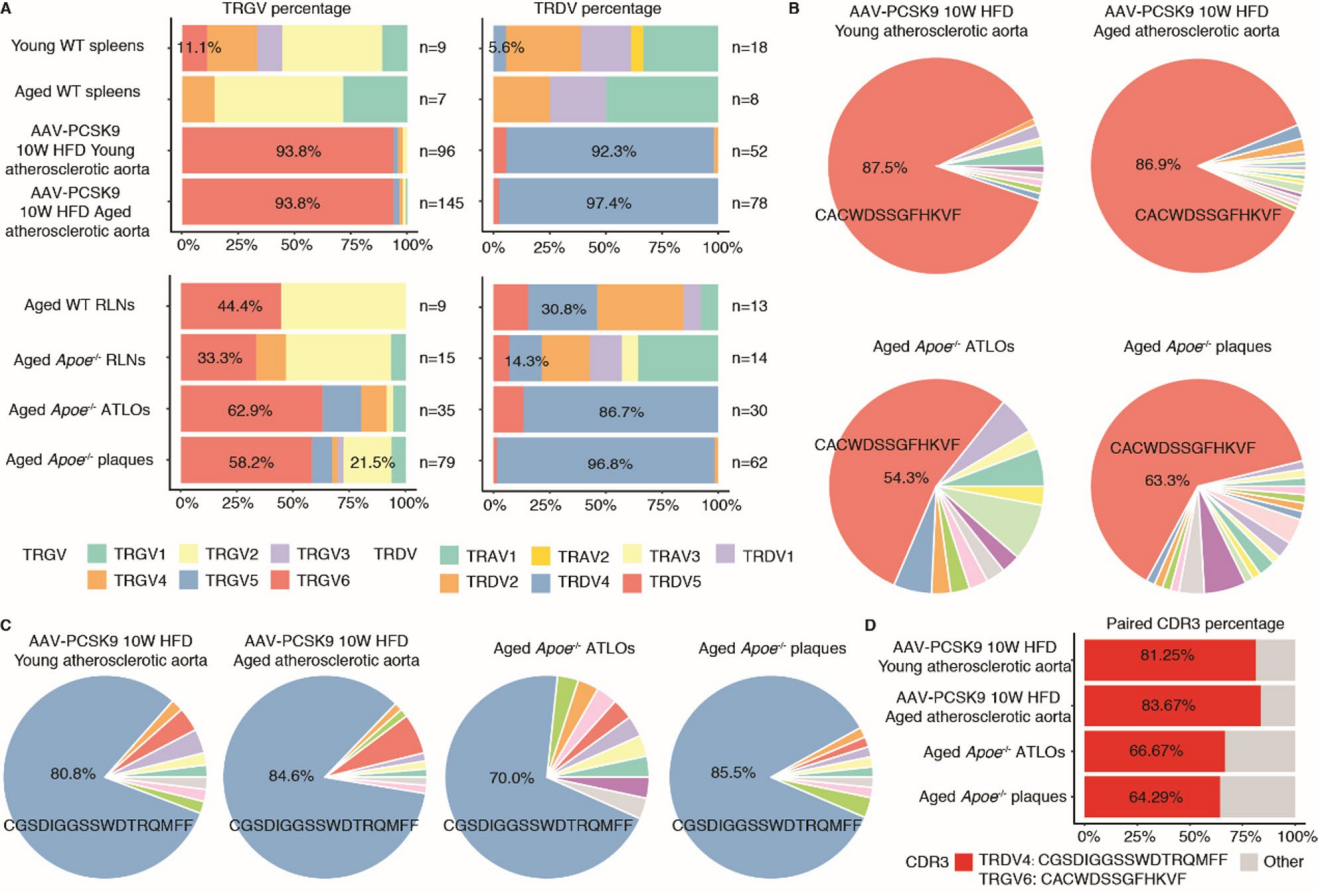
TCR repertoire analysis of mouse γδ T cells demonstrate clonal expansion of TRGV6TRDV4 γδ eduT17 cells in atherosclerotic plaque lesions and ATLOs. **A.** Composition of TRGV and TRDV gene usage in γδ T cells from studies of young and aged WT mice, as well as HFD-fed AAV-PCSK9 atherosclerotic mice, alongside aged WT and *Apoe*^−/−^ mice fed a normal chow-diet. Distinct colors represent different TRGV or TRDV subfamilies. Pie charts illustrate the percentage of unique CDR3 amino acid sequence of γ (**B**) and δ (**C**) chains in γδ T cells from young and aged WT mice and HFD-fed AAV-PCSK9-induced atherosclerotic mice, and aged WT and *Apoe*^*−/−*^ mice maintained on a normal chow-diet. The proportions of the specific CDR3 sequences TRGV: CACWDSSGFHKVF and TRDV: CGSDIGGSSWDTRQMFF are annotated for each group. Different colors denote unique CDR3 aa sequences. **D.** Percentage of γδ T cells with paired γδ TCR co-expressing the CDR3 sequences TRGV: CACWDSSGFHKVF and TRDV: CGSDIGGSSWDTRQMFF across each experimental group

Given the paucity of data on the γδ TCR repertoire, the mechanisms underlying TCR diversity and antigen-driven clonal expansion of γδ T cells in atherosclerotic pathogenesis is of interest but their clonal expansion has not been studied in either mouse or human atherosclerosis. To address this question, we employed the TRUST4 algorithm to systematically reconstruct γδ TCR sequences from conventional 5’ scRNA-seq datasets without V(D)J enrichment [44] (Fig. 1D). Application of this approach revealed that TRGV6 predominated in γδ T cells within atherosclerotic plaques in both AAV-PCSK9-induced and *Apoe*^−/−^ mouse models, as well as in ATLOs in aged *Apoe*^−/−^ mice (Fig. 4A). This is consistent with the elevated proportion of γδ eduT17 cells in plaques and ATLOs (Fig. 3C, D) and with previous observations that the majority of γδ T17 cells in murine peripheral tissues utilize Vγ4 or Vγ6 gene segments [46]. Similarly, analysis of TRDV chains disclosed substantial diversity between atherosclerotic plaques and SLOs, with TRDV4 exhibiting the highest frequency in atherosclerotic plaques and ATLOs (Fig. 4A). To further explore the role of CDR3 regions, which is required for antigen recognition in humans [53], we assessed its diversity in our datasets. Our results identified CGSDIGGSSWDTRQMFF and CACWDSSGFHKVF as the dominant CDR3 sequences in the γ and δ chains, respectively (Fig. 4B, C, Additional file 2: Table S5). Additionally, in the γδ T cell populations with paired γ and δ chains from atherosclerotic plaques and ATLOs, the majority of cells expressed the aforementioned CDR3 amino acid sequences derived from the Vγ6δ4 (TRGV6TRDV4) subset (Fig. 4D). This specific paired CDR3 clonal expansion was also demonstrated in *Staphylococcus aureus* skin infection [54]. Collectively, this data indicates their specific recruitment to plaques and their clonal expansion but whether clonal expansion is due to hyperactivation of the clonally expanded γδ T cells alone or dependent on recognition of endogenous autoantigens remains an open question that deserves to be investigated in the future.

### Multi-dataset scRNA-seq integration reveals functional diversity of γδ T cells in human atherosclerosis

To investigate the functional diversity of γδ T cells in human atherosclerotic plaques, we adopted the same strategy by integrating multi-tissue scRNA-seq datasets for analysis (Additional file 1: Fig. S7A). The lack of paired scRNA-seq and scTCR-seq presents challenges in clearly distinguishing γδ T cells from conventional T cells and NK cells in human scRNA-seq data [55, 56]. After re-clustering T cell populations, we found one separate cluster, i.e. the Vδ2Vγ9 cell population *(TRDV2*^+^*TRGV9*^+^) and scattered *TRDC*^+^ cells within conventional αβ T cell and NK cell populations (Additional file 1: Fig. S7B-D). To fully characterize the diversity of human γδ T cells in atherosclerosis, we adopted the strategy by extracting γδ T cells from (i) the Vδ2Vγ9 cell cluster and (ii) T cells with the expression of *TRDC*, which is exclusively expressed in γδ T cells. To further delineate γδ T cells and decipher their heterogeneity in humans, we further integrated our dataset with referenced FACS-sorted γδ T cell scRNA-seq data obtained from multiple tissues and SLOs in adult humans [43] (Additional file 1: Fig. S7A, Additional file 2: Table S6). We employed UMAP visualization and iLISI score to assess the effectiveness of batch effect correction in integrated human γδ T cells. The UMAP demonstrated that cells from different datasets were well integrated, in contrast to the distinct batch-specific clustering observed in uncorrected data (Additional file 1: Fig. S8A, B). Additionally, the iLISI scores were significantly higher in the integrated dataset compared to those without batch effect correction, suggesting a successful reduction of batch effects (Additional file 1: Fig. S8C). γδ T cells from human atherosclerotic plaques were distinctly separated from those in normal aorta and other tissues (Additional file 1: Fig. S8D), indicating that the disease condition may significantly modulates their gene expression profiles or selective enrich different γδ T cell subtypes in human atherosclerosis.

**Fig. 5:**
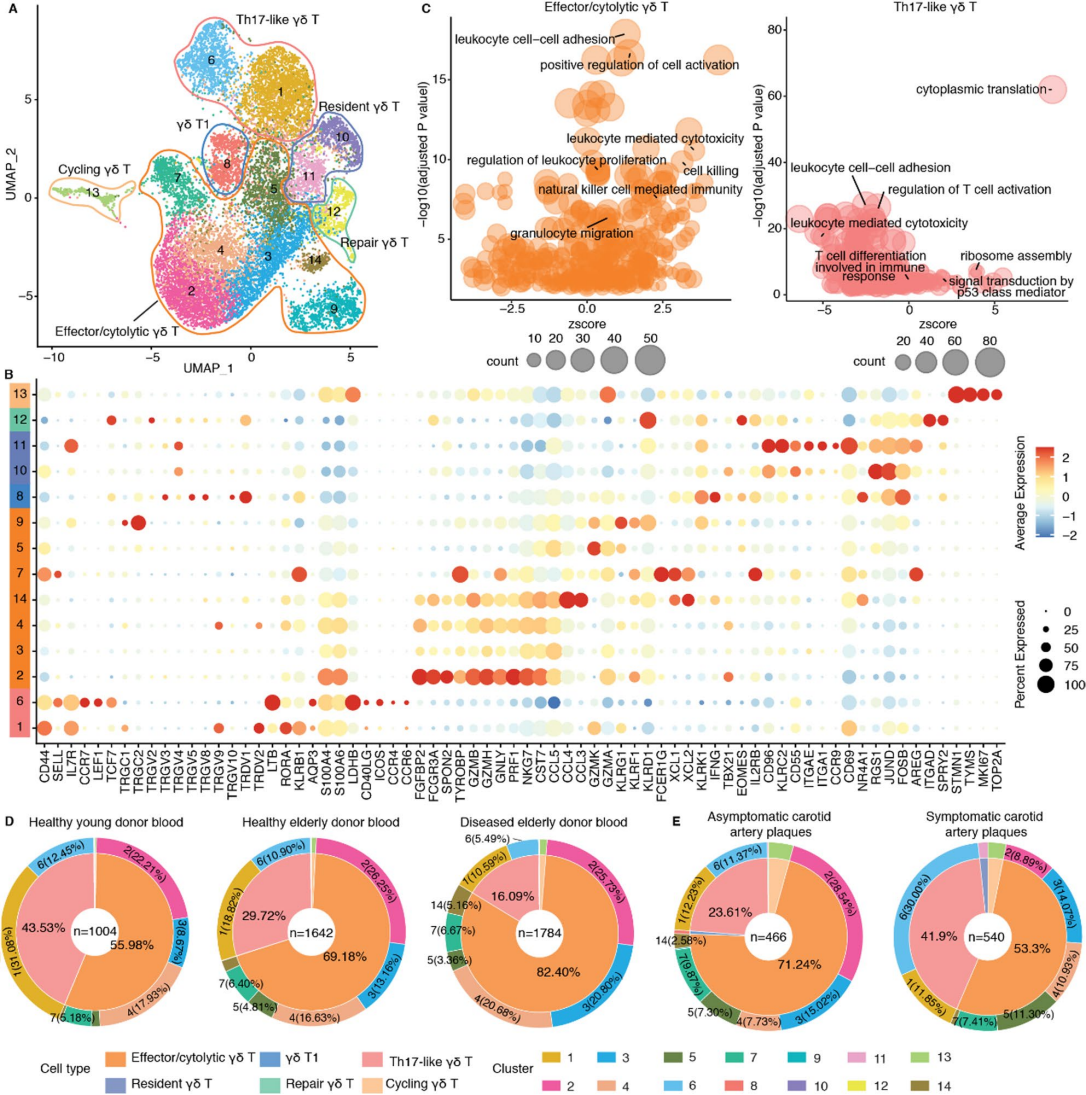
Multi-sample scRNA-seq integration uncovers and indicates functional difference of effector/cytolytic and Th17-like γδ T cells in human atherosclerosis. **A **UMAP plot visualizes γδ T cell subtypes across multiple human tissues in integrated scRNA-seq datasets. **B** Average gene expression of cell type-specific markers in each cluster of γδ T cells. **C **Bubble plot depicts GO pathway enrichment in effector/cytolytic and Th17-like γδ T cells. Dot size represents the count of genes enriched in each pathway. The X axis (zscore) indicates the biological process in one particular pathway is either decreased (negative value) or increased (positive value). Cell percentage of γδ T cell type or cluster across different human blood **D **and carotid artery plaque **E **samples. The inner circle represents different γδ T cell subtypes. The outer circle represents cell clusters

Fourteen transcriptome-specific clusters (C1-C14) were identified in the multiple tissue integration data of human γδ T cells (Fig. 5A). Clusters exhibiting a naïve phenotype were absent (Fig. 5B). C1 and C6 were characterized by the expression of *RORA*, *LTB*, *IL7R*, *S100A4*, *S100A6*, *CCR6*, and *AQP3*, which were representative markers of Th17 cells and had been implicated in identification of Th17-like γδ T cells in human blood and thymocytes [57, 58] (Fig. 5B). However, unlike mouse γδ T17 cells, human Th17-like γδ T cells did not exhibit detectable expression of *IL17A* or *IL17F*, two critical effector cytokines of γδ T17 cells (Additional file 1: Fig. S4A). Several clusters (C2, C3, C4, C5, C7, C9, C14) showed high expression levels of NK or cytotoxic genes, such as *FCGR3A*, *GNLY*, *NKG7*, *CCL5*, *PRF1*, *GZMH*, *CST7*, and *CCL4*, characteristic of effector and cytolytic γδ T cells (effector/cytolytic γδ T cells) (Fig. 5B). Elevated NK-like and cytotoxic signature scores in these clusters suggest a cell killing capacity and functional resemblance of cytotoxic αβ T cells or NK cells (Additional file 1: Fig. S8D), potentially exacerbating pro-atherogenic functions in human atherosclerosis [59]. C10 and C11 were identified as resident γδ T cells, expressing classical conventional αβ TRM cell markers such as *CD69*, *ITGAE*, and *ITGA1*. Other genes typically associated with TRM cells, including *JUND*, *FOSB*, *RGS1*, and *FOS* also exhibited high expression levels [60] (Fig. 5B). C12 was characterized by cells with higher expression of tissue repair phenotype genes (*AREG*, *ITGAD*), which had previously been reported in spleen [43]. C13 was defined as cycling γδ T cells, marked by high expression of cell cycle-related genes. C8 highly expressed *IFNG* and *TBX21*, which resembled γδ T1 cells. Pathway enrichment analysis showed that effector/cytolytic γδ T cells significantly expressed leukocyte adhesion-, activation-, and proliferation-related genes (Fig. 5C). In addition, leukocyte-mediated cytotoxicity and cell killing were also significantly expressed. In contrast, Th17-like γδ T cells exhibited a marked decrease in these pathways (zscore < 0), whereas pathways related to cytoplasmic translation and ribosome assembly were increased, reflecting divergent transcriptome patterns and potentially functionally relevant profiles compared to γδ T17 cells as observed in mice (Fig. 3B). Further, significant DEGs in γδ T1 cells were expressed in leukocyte adhesion, mononuclear cell proliferation, mononuclear cell differentiation, and cytokine production. Tissue-resident and tissue-repair cell clusters also may play critical roles in modulating leukocyte functions, as multiple pathways were significantly expressed in these two clusters (Additional file 1: Fig. S9A).

### Compositional diversity of γδ T cell subtypes in different human tissues

To define the potential characteristics of γδ T cells in atherosclerosis, we analyzed the composition and relative abundance of different T cell subtypes across different datasets. γδ T cells constituted approximately 1–5% of total T cells across different samples (Additional file 1: Fig. S9B), which consistent with the prior staining results in human atherosclerotic plaques [61]. In addition, no significant differences observed under different disease conditions. The proportion of γδ T cells in human atherosclerosis plaques was far less than their counterparts in mouse plaques (Additional file 1: Fig. S3D), highlighting a significant difference in γδ T cells in human versus mouse atherosclerosis. Significant variability in γδ T cell composition was observed across different adult human tissues (Additional file 1: Fig. S9C), consistent with prior publications [43], supporting the appropriate integration and cell subtype classification of current analysis. In human peripheral blood, aging and disease were associated with a decline in Th17-like γδ T cells and a concomitant increase in effector/cytolytic γδ T cells, suggesting that the dynamic shift in these subpopulations may associated with aging and disease (Fig. 5D). In contrast, human atherosclerotic plaques were predominated by effector/cytolytic and Th17-like γδ T cells (Fig. 5E, Additional file 1: Fig. S9D-F). More importantly, compared to asymptomatic carotid plaques, Th17-like γδ T cells accounted for 41.9% of γδ T cells in symptomatic plaques, nearly 2-fold to the proportion observed in asymptomatic plaques (Fig. 5E). The proportion of effector/cytolytic γδ T cells decreased from 71.24% to 53.3% in symptomatic plaques. Further analysis revealed only minor differences were detected in atherosclerotic plaques of different locations (Slysz et al.’s and Alsaigh et al.’s studies) (Additional file 1: Fig. S9D, E). However, differences of γδ T cell compositions in different arteries, which absent of plaque calcification or stenosis, were also observed (Hu et al.’s study) (Additional file 1: Fig. S9F). Collectively, these findings underscore the heterogeneity of γδ T cell compositions across human tissues and disease conditions.

### Effector/cytolytic and Th17-like γδ T cells may be differentially associated with plaque vulnerability

**Fig. 6:**
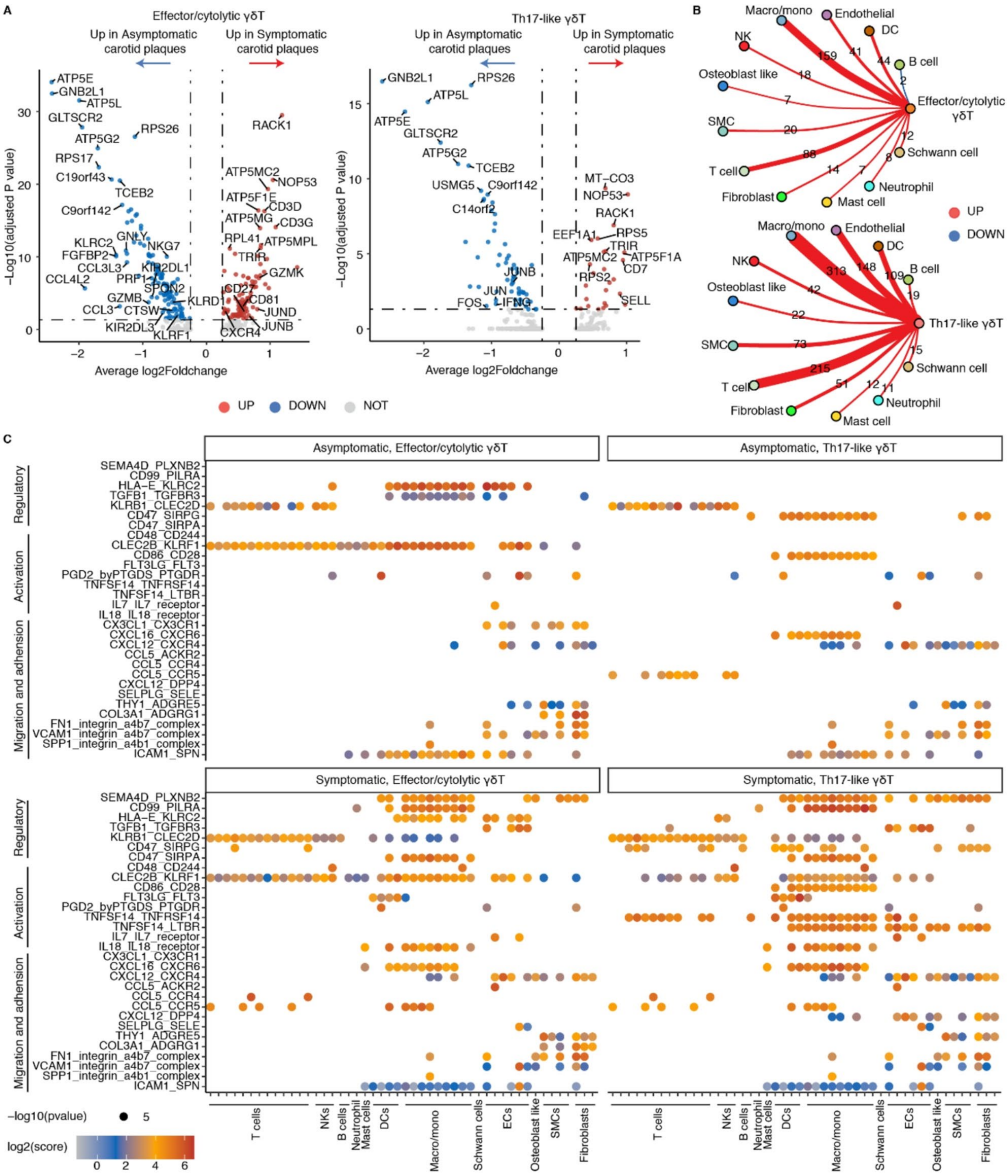
Gene expression and cell-cell communication profiles of human γδ T cells in asymptomatic versus symptomatic carotid plaques. **A**. Volcano plot showing DEGs in effector/cytolytic and Th17-like γδ T cells between asymptomatic and symptomatic carotid plaques. **B**. Circular plots illustrating differences in the number of predicted cell-cell interactions between effector/cytolytic or Th17-like γδ T cells and other cell types in asymptomatic versus symptomatic carotid plaques. The numbers represent the difference in interaction counts. Red connecting lines denote a higher number of in interactions in symptomatic plaques, whereas blue lines indicate a higher number of interactions in asymptomatic plaques. **C.** Dot plot showing representative ligand-receptor pairs between effector/cytolytic or Th17-like γδ T cells and other immune and non-immune cell types in asymptomatic and symptomatic carotid plaques. Dot size represents the statistical significance (-log10 *P* value), and color intensity reflects interaction strength (log2 interaction score). NK, natural killer cell; DC, dendritic cell; EC, endothelial cell; Macro/momo, macrophage and monocyte; SMC, smooth muscle cell

To examine a potential association between γδ T cells and clinical manifestations of atherosclerosis, we compared patients with asymptomatic and symptomatic plaques. We first analyzed DEGs in effector/cytolytic and Th17-like γδ T cells between the two clinically important plaque types. Effector/cytolytic γδ T cells showed a greater number of DEGs than Th17-like γδ T cells, with most DEGs in both subsets related to mitochondrial or ribosomal functions (Fig. 6A). Notably, cytotoxic and cytokine-associated genes, including *GZMB*,* NKG7*,* GNLY*,* PRF1*,* CCL3*,* CCL4L2*, and *CCL3L3*, were highly expressed in effector/cytolytic γδ T cells in asymptomatic plaques, suggesting a pronounced pro-atherogenic transcriptional profile of this subset in asymptomatic plaques. Pathway enrichment also supported the differences in gene expression profiles (Additional file 1: Fig. S10A). However, given the increased abundance of effector/cytolytic γδ T cells in asymptomatic plaques, these findings raised the possibility that additional γδ T cell features - beyond intrinsic cytotoxic programs - may contribute to plaque vulnerability (as expected from a multifactorially impacted chronically inflamed tissue).

We then examined whether effector/cytolytic and Th17-like γδ T cells might influence clinical manifestations through interactions with other immune and non-immune cells. Cell-cell communication analysis revealed extensive interactions between γδ T cell subsets and multiple other cell types, with a higher overall interaction numbers observed in symptomatic plaques (Fig. 6B, Additional file 1: Fig. S10B). The most pronounced differences of interaction patterns between different plaque vulnerability status were found in macrophages/monocytes, T cells, endothelial cells, and dendritic cells (DCs), with Th17-like γδ T cells displaying greater interaction diversity than effector/cytolytic γδ T cells (Fig. 6B). Representatively significant ligand–receptor pairs were further categorized into pathways mediating cell migration and adhesion, activation, and suppression (Fig. 6C).

Overall, γδ T cells in symptomatic plaques exhibited a marked expansion of predicted interactions, particularly within the Th17-like γδ T cells. Notably, Th17-like γδ T cells exhibited the most extensive interaction network as characterized by strong enrichment of activation-related communication networks with both immune and non-immune cell types versus effector/cytolytic γδ T cells, especially with macrophages and DCs. This interaction pattern is consistent with an amplified pro-inflammatory and tissue-remodeling communication program during plaque destabilization. Symptomatic plaques showed an accumulation of DCs and T cells, particularly CD8 effector and memory T cells (Additional file 1: Fig. S10C). The FLT3LG-FLT3 interaction was exclusively observed between γδ T cells and DCs in symptomatic plaques (Fig. 6C), which was even stronger in Th17-like γδ T cells. This interaction may promote the accumulation, proliferation, and antigen presentation of DCs, as demonstrated by FLT3L treatment to dramatically expand the number of DCs in prior studies [62]. The accumulation and augmented cross-presentation capability of DCs may further promote CD8 T cell activation. Different from the symptomatic plaques, macrophages, and within their subtypes particularly TREM2^+^ macrophages, were the major enriched immune cells in asymptomatic plaques. TREM2^+^ macrophages had been shown previously to restrict necrotic core formation and plaque growth by modulating lipid uptake and clearance of cell debris and dead cells [63, 64]. Of note, the CD47 in both effector/cytolytic and Th17-like γδ T cells showed pronounced interaction with SIRPα (*SIPRA*) on macrophages/monocytes in symptomatic plaques (Fig. 6C), which may trigger the “don’t eat me” signaling cascade to dampen the phagocytic activity of macrophages [65].

Collectively, although effector/cytolytic γδ T cells display a pro-atherogenic transcriptional phenotype in asymptomatic plaques, the vulnerable plaque status appears to strongly reshape the γδ T cell communication. Symptomatic lesions are characterized by globally enhanced and diversified γδ T cell interactions, with Th17-like γδ T cells exhibiting a broader and more inflammation-oriented communication landscape than effector/cytolytic γδ T cells, supporting their potential contribution to immune response amplification and plaque progression in symptomatic atherosclerosis.

### TCR repertoire of γδ T cells in human atherosclerosis

We further analyzed the TCR repertoire of γδ T cells in human carotid atherosclerotic plaques, categorized by symptomatic status or plaque stability. Our findings revealed a significant enrichment of TRBV and TRAV gene segments relative to TRGV and TRDV segments within these plaques (Additional file 1: Fig. S11, Additional file 2: Table S7), aligning with our observation of a reduced prevalence of γδ T cells in human atherosclerotic plaques (Additional file 1: Fig. S9B). Although the number of reconstructed γδ TCR sequences were limited, we identified several TRDV and TRGV clones exhibiting substantial clonal expansion. Notably, the CDR3 amino acid sequences of these clones were not conserved across patients (Additional file 2: Table S8), indicating substantial diversity. This finding contrasts markedly with the considerable overlap of CDR3 aa sequences observed in atherosclerotic plaques from mice, highlighting species-specific differences in γδ T cell repertoire dynamics.

## Discussion

**Fig. 7:**
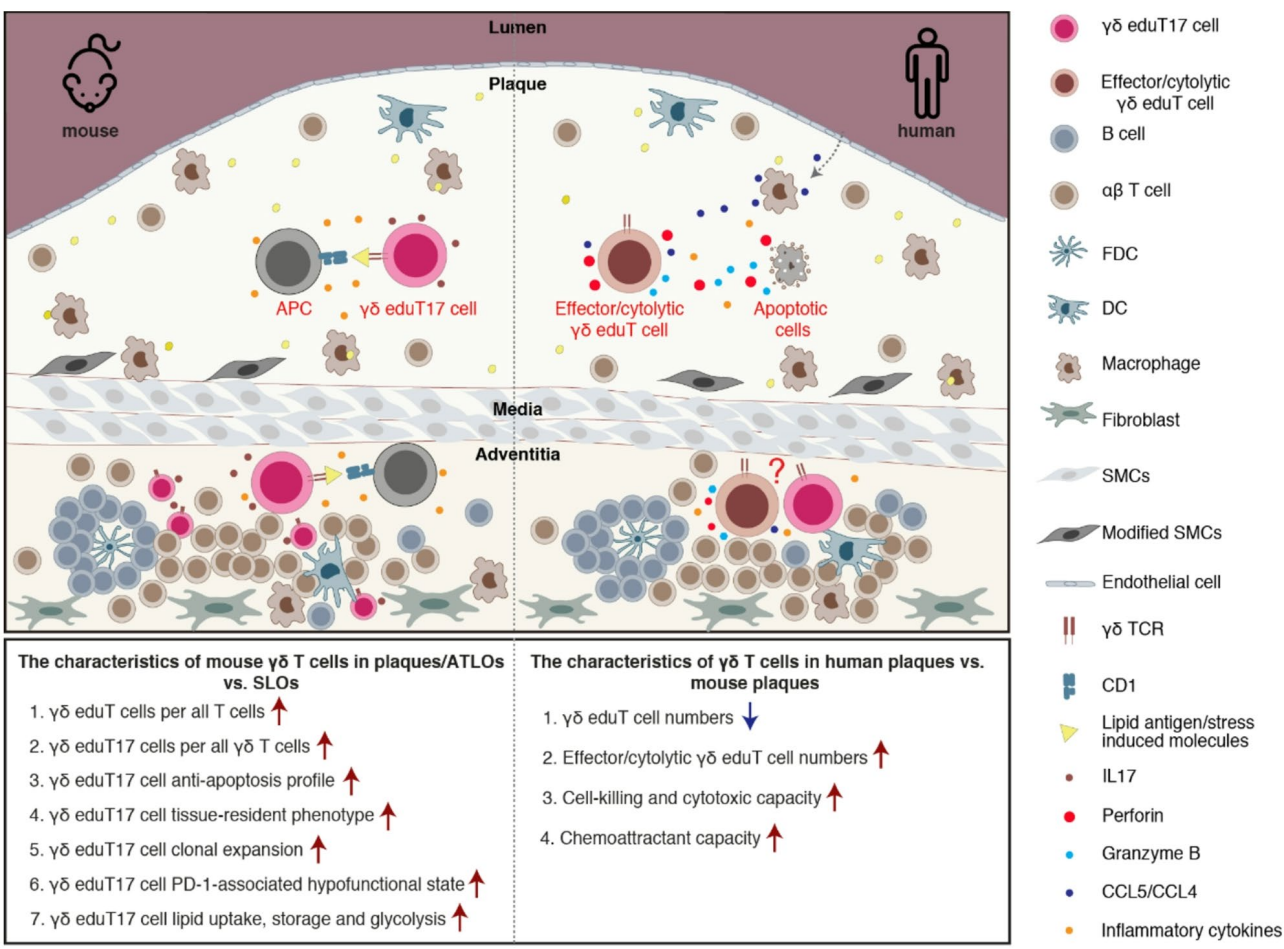
Characteristics of γδ T cells in mouse versus human atherosclerotic plaques and ATLOs. Using a three-pronged approach, we observed increases of γδ T cell density among total T cells in mouse atherosclerotic plaques and ATLOs, with preferential localization in ATLOs over plaques. Proinflammatory IL17-producing γδ T17 cells were the dominant subtype in both tissues. A γδ T17 subtype expressing paired Vγ6Vδ4 TCR chains with identical CDR3 regions exhibited clonal expansion in both plaques and ATLOs in mice. APCs may support γδ T cell activation and clonal expansion in atherosclerosis through two non-mutually exclusive mechanisms: non-classical antigen presentation of lipid antigens or stress-induced molecules (e.g., via CD1 molecules), and cytokine-mediated inflammatory stimulation (e.g., IL1β and IL23). The inflammatory atherosclerotic microenvironment trains γδ T17 cells to modify their anti-apoptotic, tissue-resident-like, hypofunctional-like and metabolic program phenotypes. We designated the educated γδ T17 cells in plaques and ATLOs as γδ eduT17 (edu for educated). In contrast, γδ T cell numbers were scarce in human plaques, where effector/cytolytic γδ eduT cells with cytotoxic and cell-killing profiles predominated. Human ATLOs were included in the human panel based on prior studies with little information on their γδ T cell subtypes and therefore were labeled with a question mark. γδ eduT17 or effector/cytolytic γδ eduT cells represent educated γδ T cell subtypes in atherosclerotic plaques and ATLOs. The effector/cytolytic γδ eduT cells also release CCL5/CCL4 chemokines with multiple functions including recruitment of macrophages to inflamed tissues via their receptor on macrophages. FDC, follicular dendritic cell; DC, dendritic cell; SMC, smooth muscle cell; APC, antigen-presenting cells

Our data described above support several conclusions: The majority of all mouse γδ T cells in diseased arterial walls localize in ATLOs in aged hyperlipidemic mice with predominance of the γδ eduT17 subtype; γδ eduT17 cells with paired Vγ6Vδ4 TCR chains and identical CDR3 regions in mouse plaques and ATLOs are clonally expanded; plaques and ATLOs educate the γδ T17 cells to modify their tissue-resident, anti-apoptotic, hypofunctional-like and metabolic reprograming phenotypes; in contrast, human plaques are home to only few γδ T cells which, however, were dominated by an effector/cytolytic γδ eduT cell phenotype expressing the perforin/granzyme and CCL4/CCL5 (Fig. 7). Our data suggest different roles of these enigmatic and rare family of T cell subtypes in mouse versus human atherosclerosis.

We observed that the majority of mouse γδ T cells within the diseased arterial wall reside in ATLOs raising the question as to the mechanisms of their recruitment and their specific roles in ATLOs versus plaques. Several possibilities deserve attention: First, unlike plaques, stage III ATLOs have a prominent B cell compartment with activated B cell follicles containing germinal centers with follicular dendritic cells [66]. Spatial transcriptomic analysis revealed that the majority of ATLO γδ T cells reside within the border zone between T cell areas and B cell follicles, indicating that they may be related to facilitation of germinal center formation to participate in the control of affinity maturation of antibodies. This anatomical location may be related to previously reported impaired high affinity antibody formation observed in *TCRδ*^−/−^ mice after vaccination [24]. This body of data including its anatomical location is consistent with the possibility that γδ T cells in atherosclerosis also may have significant roles in adaptive B cell functions. Further studies will be needed to demonstrate the role of γδ T cells in the regulation of adaptive B cell immunity and specifically their involvement in ATLO affinity maturation of B cell receptors in germinal centers during atherosclerosis progression.

Mouse γδ eduT17 cells with paired Vγ6Vδ4 TCR chains and identical CDR3 regions were observed to be clonally expanded in plaques and ATLOs. This finding raises the important question where clonal expansion of the diseased arterial wall-residing γδ eduT17 cells occurs, i.e. in ATLOs or in atherosclerotic plaques or both. Regardless of this unanswered question, γδ T17 cells are constitutively proinflammatory and we suggest that they may contribute to the highly inflammatory tissue environment of ATLOs after their education to acquire the γδ eduT17 phenotype [23]. It is well established for various types of cancer and autoimmune diseases that γδ T17 cells accomplish very different impacts in these diseases: The highly proinflammatory γδ T17 have been reported to promote disease progression in autoimmune diseases including inflammatory bowel diseases, rheumatoid arthritis, and psoriasis [8]. Although our current analyses do not allow definitive conclusions regarding γδ TCR repertoire in human plaques due to limitations in the available data, human γδ T cells with cytotoxic effector functions have been shown to exhibit potent antitumor effects, prompting the development of multiple immunotherapeutic strategies targeting γδ T cells for the treatment of human cancers [9–12]. If those characteristics are considered in relation to our current data, we are inclined to suggest that the overall role of the educated effector/cytolytic γδ T cells featuring a cytotoxic profile with perforin and granzyme B and CCL4/CCL5 as lead-expressed genes may contribute to plaques vulnerability.

Integration of multiple single-cell datasets allowed us to analyze the rare γδ T cells at single-cell resolution while substantially increasing overall cell numbers and statistical robustness. This strategy enabled us to identify conserved γδ T cell phenotypes across different mouse models and experimental conditions. Nevertheless, even after extensive dataset integration, the absolute number of γδ T cells derived from atherosclerotic plaques remained limited. Incorporation of additional FACS-sorted γδ T cells scRNA-seq datasets - particularly from atherosclerotic lesions – will further enhance resolution of γδ T cell subtypes and states. One potential concern of large-scale integration is that may distort clustering outcomes. However, murine γδ T cells differ fundamentally from αβ T cells in the developmental programming. A substantial fraction of γδ T cells commits to either IFN-γ or IL17 expression already during thymic development [34]. Consequently, core γδ T cell transcriptional programs are largely established prior to their appearance in peripheral tissues, providing a biological rationale for stable cross-tissue integration in our study. Consistently, our batch effect evaluations and multiple controls, such as the restriction of progenitor γδ T cell populations to the thymus, support the appropriateness of our integration strategy.

Accurate identification of human γδ T cells remains challenging in the absence of additional sc-γδ TCR-seq or CITE-seq data, particularly when using 3′-end scRNA-seq analysis platforms [56]. In our current analyses, we adopted a widely accepted strategy by extracting clustered Vγ9Vδ2⁺ γδ T cells and scattered *TRDC*^+^ non-Vγ9Vδ2⁺ γδ T cells, which represent a highly heterogeneous population and often displays transcriptional programs resembling cytotoxic CD8⁺ T cells or NK cells [43, 67]. Although *TRDC* is considered a relatively specific γδ T cell marker, we acknowledge that relying solely on *TRDC* expression may lead to underestimation of γδ T cell abundance, particularly for non-Vγ9Vδ2^+^ subsets in 3’-end scRNA-seq data. Nonetheless, this approach remains standard practice and is supported by prior studies [55, 67]. Importantly, our results are biologically consistent with prior reports. In our current integration data, γδ T cells accounted for approximately 5% of CD3^+^ T cells in healthy adult human blood, consistent with previous flow cytometry-based estimates (1–10%) [68–70]. In elderly donors, we observed a reduction of γδ T cells to ~ 2.5%, which aligns with well-documented age-associated decline in circulating γδ T cells [71]. Across carotid atherosclerotic datasets, the observed γδ T cell frequencies were also consistent with earlier staining-based studies [61]. Together, these observations support the reliability of our current integration framework and its suitability for downstream biological inference.

Through integrated analysis of multiple mouse atherosclerosis models, we identified several major γδ T cell clusters, including γδ T17, γδ T1, naïve-like γδ T cells, and NK-like γδ T cells. The transcriptional signatures of these populations closely correspond to the core γδ T cell subsets described in recent single-cell analyses of murine γδ T cell heterogeneity [46]. Extending these observations into the disease context, our analyses revealed consistent enrichment, functional education, and clonal expansion of γδ T17 cells within atherosclerotic lesions across independent models. These findings emphasize how chronic lipid-rich and inflammatory vascular environments selectively reshape γδ T17 cell gene expression profiles in atherosclerosis. For human γδ T cells, we adopted the classification framework proposed by Gray et al. [43], identifying effector/cytolytic, Th17-like, γδ T1, tissue-resident, and repair-associated γδ T cells in our current integration datasets. Although Th17-like γδ T cells were not explicitly annotated as a separate category in that study, one γδ T cell cluster displayed elevated expression of Th17-associated genes, including *IL7R*, *RORA*, *ZBTB16*, *LTB*, *S100A4*, and *KLRB1*. Notably, this transcriptional profile is highly consistent with previously reported Th17-like γδ T cell signatures in humans [57], supporting our designation of this population as Th17-like γδ T cells here.

A notable species difference emerged from our analyses. In mice, γδ T cells constituted a relatively large fraction of total T cells within atherosclerotic plaques, enabling systematic assessment of enrichment dynamics, education, and clonal expansion across different ages, tissues, and disease models. γδ T17 cells emerged as the dominant subset within atherosclerotic lesions, displaying disease-associated education and TCR clonal expansion. In contrast, human atherosclerotic plaques exhibit substantially lower frequencies of γδ T cells relative to total T cells, with dominance of effector/cytolytic phenotypes in atherosclerotic plaques. The limited cell numbers, lack of controls, and absence of paired TCR information in human datasets precluded formal assessment of γδ T cell education and clonal expansion in this setting.

Due to the nature of available mouse atherosclerosis models, disease progression is inherently coupled with age and/or duration of dietary intervention. While this limits our ability to fully disentangle these variables from disease-specific effects versus bona fide aging, our inclusion of secondary lymphoid organs from WT mice across different ages revealed that aging-associated changes are associated with increased representation of γδ T17 cells (Fig. 3C, D and Additional file 1: Fig. S5), without inducing major transcriptional reprograming (data not shown). Furthermore, γδ T17 enrichment, clonal expansion, and acquisition of the educated γδ T17 phenotype were consistently observed in the female-only dataset (AAV-PCSK9 mouse model, GSE210719) and were comparable to those in male-only atherosclerosis datasets, supporting the conclusion that the atherosclerotic inflammatory microenvironment, rather than age or sex alone, is the dominant driver of γδ T17 cell accumulation and education.

When taken together, our data provides a blueprint for further studies of the rare and still mysterious γδ T cells and their subtypes in mouse and human atherosclerosis. In particular, our data will permit to further expand the study on the roles of γδ T cells in innate versus adaptive immune responses; identify immune tolerance checkpoints; examine the mechanisms of their recruitment into the diseased arterial wall; functionally examine their tissue-resident phenotypes; and unravel mechanisms of their education programs. The mechanistic insight reported here may lead to approaches to target γδ T cells as therapeutics in atherosclerosis.

## Conclusions

Our study provides single-cell transcriptome and anatomical maps of γδ T cell subtypes in atherosclerosis, integrating scRNA-seq, scTCR-seq, and spatial transcriptomics in mouse models, together with large-scale multi-dataset integration of human atherosclerotic plaques. We reveal that murine atherosclerosis is characterized by enrichment, local education, and clonal expansion of proinflammatory γδ eduT17 cells within plaques and ATLOs. Importantly, our data indicate that the atherosclerotic microenvironments, rather than age or sex alone, are major drivers of γδ T17 cell accumulation and education. In contrast, human atherosclerotic lesions contain substantially fewer γδ T cells and are dominated by γδ T cells with effector/cytolytic transcriptional features, suggesting divergent roles of γδ T cells between species. Collectively, these findings clarify many hitherto unanswered questions regarding the potentially important roles of γδ T cells in atherosclerosis progression. Our data provide a blueprint for further investigating the roles of the enigmatic γδ T cell subtypes in atherosclerosis pathogenesis and other diseased tissue in which γδ T cells are exceedingly rare.

## Supplementary Information

Additional file 1. Fig S1. Spatial transcriptomics reveal the location of cells in advanced *Apoe*^−/−^ atherosclerotic aorta and RLNs. Fig S2. Spatial transcriptomics reveal the distribution of *Trdc*^+^ spots. Fig S3. Integration analysis of multiple γδ T cell scRNA-seq datasets of different tissues across WT and hyperlipidemic mouse models. Fig S4. scRNA-seq analysis identify γδ T cell subtypes in mouse atherosclerosis. Fig S5. Proportions of γδ T cell subtypes and clusters across diverse mouse datasets. Fig S6. Gene expression profiles of γδ T17 cells during aging in diseased aorta, SLOs and ATLOs. Fig S7. Multi-dataset scRNA-seq integration analysis of γδ T cells in human atherosclerosis. Fig S8. Heterogeneity and transcript profiling of γδ T cells in human atherosclerosis. Fig S9. Functional diversity and proportional distribution of γδ T cell subsets across various human tissues. Fig S10. Distinct features of γδ T cell subtypes in symptomatic and asymptomatic carotid plaques. Fig S11. TCR repertoire analysis of human carotid plaques using TRUST4 on bulk RNA-seq data

Additional file 2. Table S1: Information on mouse datasets for multi-sample scRNA-seq integration. Table S2: GO pathway enrichment analysis of γδ T17 cells. Table S3: GO pathway enrichment analysis of γδ T1 cells. Table S4: GO pathway enrichment analysis of NK-like γδ T cells. Table S5: Percentage of unique CDR3 aa sequences of γ and δ chains in γδ T cells of different tissues in different datasets. Table S6: Information on human datasets for multi-sample scRNA-seq integration. Table S7: Re-constructed TCR from stable/asymptomatic and unstable/symptomatic human plaques. Table S8: Re-constructed γδ TCR from stable/asymptomatic and unstable/symptomatic human plaques.

## Data Availability

All scRNA-seq datasets used here are available for download from the GEO database under the relevant accession numbers or via the links provided in the Methods section. The spatial transcriptomic sequencing data generated in this study are not publicly available as they are part of an unpublished dataset. The data are available from the corresponding author upon reasonable request.

## Abbreviations

ATLOs: Artery Tertiary Lymphoid Organs
scRNA-seq: Single-cell RNA sequencing
scTCR-seq: Single-cell T Cell Receptor sequencing
γδ T17: IL17-producing γδ T cells
γδ T1: IFNγ-producing γδ T cells
γδ eduT17: Educated γδ T17 cells
HFD: High-fat diet
ND: Normal diet
WT: Wild-type
RLN: Aorta-draining renal lymph node
RPCA: Reciprocal principal component analysis
CDR3: Complementarity-determining region 3
DEGs: Differentially expressed genes
SLOs: Secondary Lymphoid Organs
