# Supplementary material for: Fitness Training for γδ T cells in mouse and human atherosclerosis takes place in plaques and artery tertiary lymphoid organs
Source: Genome Med. 2026 Feb 24;18:26. doi: 10.1186/s13073-026-01613-1 (PMC12931092; doi:10.1186/s13073-026-01613-1)
Supplement: Supplementary file 1 — Additional file 1. Fig S1. Spatial transcriptomics reveal the location of cells in advanced Apoe−/− atherosclerotic aorta and RLNs. Fig S2. Spatial transcriptomics reveal the distribution of Trdc+ spots. Fig S3. Integration analysis of multiple γδ T cell scRNA-seq datasets of different tissues across WT and hyperlipidemic mouse models. Fig S4. scRNA-seq analysis identify γδ T cell subtypes in mouse atherosclerosis. Fig S5. Proportions of γδ T cell subtypes and clusters across diverse mouse datasets. Fig S6. Gene expression profiles of γδ T17 cells during aging in diseased aorta, SLOs and ATLOs. Fig S7. Multi-dataset scRNA-seq integration analysis of γδ T cells in human atherosclerosis. Fig S8. Heterogeneity and transcript profiling of γδ T cells in human atherosclerosis. Fig S9. Functional diversity and proportional distribution of γδ T cell subsets across various human tissues. Fig S10. Distinct features of γδ T cell subtypes in symptomatic and asymptomatic carotid plaques. Fig S11. TCR repertoire analysis of human carotid plaques using TRUST4 on bulk RNA-seq data [file 13073_2026_1613_MOESM1_ESM.docx]

**Additional file 1**

**Fitness Training for γδ T Cells in Mouse and Human Atherosclerosis Takes Place in Plaques and Artery Tertiary Lymphoid Organs**

**Zhihua Wang et al.,**

**Fig S1. Spatial transcriptomics reveal the location of cells in advanced *Apoe*^-/-^ atherosclerotic aorta and RLNs. A.** Representative images illustrate high numbers of transcripts expressed as feature/gene ­spatial heatmap scale across distinct regions. Locations are numbered in the top left corner. Location 1 represents RLN; Location 2 represents advanced atherosclerotic plaque with an adjacent ATLO; Location 4 represents atherosclerotic plaques without adventitial immune cell aggregates. Dashed line represents the border of atherosclerotic plaques or lymph node. **B.** Representative images display the distribution of major immune and non-immune cell types across distinct locations. Note the low degree of cellularity of plaques versus ATLOs.

**Fig S2. Spatial transcriptomics reveal the distribution of *Trdc^+^* spots.** Representative images illustrate *Trdc*^+^ spots across distinct regions. Locations are numbered in the top left corner. Location 1 represents RLN; Location 2, 3 and 5 represent advanced atherosclerotic plaque with an adjacent ATLO; Location 4 represents atherosclerotic plaques without adventitial immune cell aggregates. Dashed line represents the border of atherosclerotic plaques, ATLO or lymph node. The locations of these representative images are identical to the those in Figure 2D.

**Fig S3. Integration analysis of multiple** **γδ T cell scRNA-seq datasets of different tissues across WT and hyperlipidemic mouse models.** **A.** UMAP visualization depicts the distribution of γδ T cells as categorized by dataset source and tissue type. Left: Data prior to batch effect correction with batch effects. Right: Data following batch effect correction reveals improved integration. Different colors represent different datasets. **B.** UMAP visualization depicts the distribution of γδ T cells as categorized by tissue type. Different colors represent different tissues. **C.** Distribution of the iLISI score for each dataset pre- and post-batch effect correction. High iLISI score represent cells from different batches are well-mixed. Each dot represents each single cell. Wilcoxon rank sum test was used to compare the difference of iLISI score pre- and post-batch effect correction in each dataset. **D.** Proportion of γδ T cells relative to the total T cells across different groups. Colors indicate distinct datasets, consistent with the color used for each dataset in panel A. Column borders denote different tissues. Dashed line with a large gap represents samples from atherosclerotic plaques and diseased aortas; Dashed line with a small gap indicates ATLOs; Solid line corresponds to SLOs and blood samples.

**Fig S4. scRNA-seq analysis identify γδ T cell subtypes in mouse atherosclerosis.** **A.** Average gene expression of cell type-specific markers in each cluster. Dot size indicates the percentage of cells expressed one certain gene. Color represents the gene expression level. **B.** Composition of cell cycle phases (G1, S, and G2/M) within each γδ T cell cluster. **C.** Bubble plot illustrating GO pathway enrichment in NK-like γδ T cells. Dot size represents the number of genes highly expressed in each pathway. The X axis (zscore) reflects the likelihood of decreased (negative value) or increased (positive value) biological processes within a given pathway.

**Fig S5. Proportions of γδ T cell subtypes and clusters across diverse mouse datasets. A.** Proportions of γδ T cell subtypes from the study by Li et al., which characterized the landscape of FACS-sorted γδ T cells derived from the thymus, peripheral lymph nodes, and spleen of C57BL/6 mice. **B.** Proportions of γδ T cell subtypes from the study by Sharma et al., which investigated T_reg_ cells during atherosclerosis regression in AAV-PCSK9-induced atherosclerotic mouse models. Proportions of γδ T cell subtypes from the study by Pan et al., which utilized SMC-lineage tracing mice with *Apoe*^−/−^ (**C**) or *Ldlr*^−/−^ (**D**) genetic backgrounds for analysis. **E.** Proportions of γδ T cell subtypes from the study by Cochain et al., which examined *Ldlr*^−/−^ mice at various ages.

**Fig S6. Gene expression profiles of γδ T17 cells during aging in diseased aorta, SLOs and ATLOs**. **A.** Anti-apoptotic module scores of Vγ4^+^ and Vγ6^+^ γδ T17 cells in AAV-PCSK9-induced and normal chow-diet-fed aged *Apoe*^−/−^ atherosclerotic mouse models across different tissues. Kruskal-Wallis rank sum test was used to calculate the significance of differences among groups. **B.** Average gene expression of anti-apoptosis associated genes of Vγ6^+^ γδ T17 cells across young WT mice lymph nodes and skin, AAV-PCSK9-induced and normal chow-diet-fed aged *Apoe*^−/−^ atherosclerotic mouse models. **C.** Average gene expression of tissue-resident T cell associated genes between Vγ4^+^ and Vγ6^+^ γδ T17 cells in AAV-PCSK9-induced and normal chow-diet-fed aged *Apoe*^−/−^ atherosclerotic mouse models across different samples. **D.** Average gene expression of tissue-resident associated genes of Vγ6^+^ γδ T17 cells across young WT mice lymph nodes and skin, AAV-PCSK9-induced and normal chow-diet-fed aged *Apoe*^−/−^ atherosclerotic mouse models. **E.** Violin plots display the differences of metabolic gene signature scores across different tissues in the Tyrrell et al. study. **F.** Violin plots display the differences of metabolic gene signature scores across different tissues in the Wang et al. study. Kruskal-Wallis rank sum test was used to calculate the significance of differences among groups. **G.** Violin plots display the differences of metabolic gene signature scores between Vγ4^+^ and Vγ6^+^ γδ T17 cells across different tissues in the Tyrrell et al. study. Wilcoxon rank sum test with continuity correction was used for statistical analysis between two groups. FAO, fatty acid oxidation; OXPHOS, oxidative phosphorylation.

**Fig S7. Multi-dataset scRNA-seq integration analysis of γδ T cells in human atherosclerosis**. **A.** Schematic illustration of the integration strategy for scRNA-seq data derived from multiple human tissue datasets to analyze γδ T cells in human atherosclerosis. **B.** UMAP visualization of T cell subtypes following multi-dataset and multi-tissue scRNA-seq integration. MAIT: Mucosal-associated invariant T cell; ISG^+^: interferon-stimulated gene positive cells; Tcmp: Central Memory precursors; NK: Natural killer cells. **C.** Gene expression level of T cell receptor delta constant (TRDC) across different T cell subtypes. **D.** Average gene expression of cell type-specific markers in each T/NK cluster. Dot size indicates the percentage of cells expressed one certain gene. Color represents the gene expression level.

**Fig S8. Heterogeneity and transcript profiling of γδ T cells in human atherosclerosis. A.** UMAP visualization depicts the distribution of γδ T cells as categorized by dataset source and tissue type. Left: Data prior to batch effect correction, exhibiting uncorrected batch effects. Right: Data following batch effect correction, demonstrating improved integration. Different colors represent different datasets. **B.** UMAP visualization depicts the distribution of γδ T cells as categorized by tissue type. Different colors represent different tissues. **C.** Distribution of the iLISI score for each dataset pre- and post-batch effect correction. Wilcoxon rank sum test was used to compare the difference of iLISI score pre- and post-batch effect correction in each group. **D**. Principal component analysis (PCA) showing the distribution of different tissues across different datasets. **E**. Comparison of NK signature and cytotoxic signature module scores across different γδ T cell clusters. Kruskal-Wallis rank sum test was used to calculate the significance of differences.

**Fig S9. Functional diversity and proportional distribution of γδ T cell subsets across various human tissues. A**. Bubble plot displays the GO pathway enrichment in γδ T1, resident, and repair γδ T cell subsets. The dot size represents the relative count of genes enriched in each pathway. The X axis (zscore) indicates the biology process in one particular pathway is either decreased (negative value) or increased (positive value). **B.** The proportion of γδ T cells relative to total T cells across different conditions in multiple datasets. If the data were normally distributed, a one-way Anova was used to compare differences between more than two groups. Otherwise, Wilcoxon rank sum test (2 groups) or Kruskal-Wallis rank sum test (more than 2 groups) was used to calculate the significance of differences. Each dot represents one sample from different conditions. The color represents to different datasets. The cell percentage of each γδ T cell subtype or each cluster in the studies by Gray et al. (**C**), Alsaigh et al. (**D**), Slyz et al. (**E**), and Hu et al. (**F**). The inner circle represents different γδ T cell subtypes. The outer circle represents the cell clusters.

**Fig S10. Distinct features of γδ T cell subtypes in symptomatic and asymptomatic carotid plaques. A.** Gene set enrichment analysis (GSEA) of effector/cytolytic and Th17-like γδ T cells between human symptomatic and asymptomatic carotid plaques. NES: normalized enrichment score. B. Bar plot showing the predicted numbers of cell-cell interactions between different γδ T cell subsets and other cell types in symptomatic and asymptomatic carotid plaques. **C**. Differences in the relative proportions of cell types between symptomatic and asymptomatic carotid plaques. Red color represents cell types enriched in asymptomatic carotid plaques, whereas blue color represents cell types enriched in symptomatic carotid plaques.

**Fig S11. TCR repertoire analysis of human carotid plaques using TRUST4 on bulk RNA-seq data.** TRUST4 was employed to reconstruct the TCR repertoire from bulk RNA-sequencing data of human carotid plaques, comparing stable/asymptomatic and unstable/symptomatic samples. Histograms illustrate the distribution of reconstructed TCR chains in human carotid plaques, categorized by symptomatic status (left panel) or plaque stability (right panel). TRAV: T cell receptor alpha variable; TRBV: T cell receptor beta variable; TRGV: T cell receptor gamma variable; TRDV: T cell receptor delta variable.
